# Recent Advances in Stretchable and Wearable Capacitive Electrophysiological Sensors for Long-Term Health Monitoring

**DOI:** 10.3390/bios12080630

**Published:** 2022-08-11

**Authors:** Hadaate Ullah, Md A. Wahab, Geoffrey Will, Mohammad R. Karim, Taisong Pan, Min Gao, Dakun Lai, Yuan Lin, Mahdi H. Miraz

**Affiliations:** 1 School of Materials and Energy, University of Electronic Science and Technology of China, Chengdu 610054, China; 2 State Key Laboratory of Electronic Thin Films and Integrated Devices, University of Electronic Science and Technology of China, Chengdu 610054, China; 3 Institute for Advanced Study, Chengdu University, Chengdu 610106, China; 4 School of Mechanical, Medical and Process Engineering, Faculty of Engineering, Queensland University of Technology, George St Brisbane, GPO Box 2434, Brisbane, QLD 4001, Australia; 5 Center of Excellence for Research in Engineering Materials (CEREM), Deanship of Scientific Research (DSR), King Saud University, Riyadh 11421, Saudi Arabia; 6 K.A. CARE Energy Research and Innovation Center, Riyadh 11451, Saudi Arabia; 7 Biomedical Imaging and Electrophysiology Laboratory, School of Electronic Science and Engineering, University of Electronic Science and Technology of China, Chengdu 610054, China; 8 Medico-Engineering Corporation on Applied Medicine Research Center, University of Electronic Science and Technology of China, Chengdu 610054, China; 9 School of Computing and Data Science, Xiamen University Malaysia, Bandar Sunsuria, Sepang 43900, Malaysia; 10 School of Computing, Faculty of Arts, Science and Technology, Wrexham Glyndŵr University, Wrexham LL112AW, UK

**Keywords:** wearable sensors, electrophysiological sensors, capacitive sensors, health monitoring, flexible electrodes, long-term health monitoring

## Abstract

Over the past several years, wearable electrophysiological sensors with stretchability have received significant research attention because of their capability to continuously monitor electrophysiological signals from the human body with minimal body motion artifacts, long-term tracking, and comfort for real-time health monitoring. Among the four different sensors, i.e., piezoresistive, piezoelectric, iontronic, and capacitive, capacitive sensors are the most advantageous owing to their reusability, high durability, device sterilization ability, and minimum leakage currents between the electrode and the body to reduce the health risk arising from any short circuit. This review focuses on the development of wearable, flexible capacitive sensors for monitoring electrophysiological conditions, including the electrode materials and configuration, the sensing mechanisms, and the fabrication strategies. In addition, several design strategies of flexible/stretchable electrodes, body-to-electrode signal transduction, and measurements have been critically evaluated. We have also highlighted the gaps and opportunities needed for enhancing the suitability and practical applicability of wearable capacitive sensors. Finally, the potential applications, research challenges, and future research directions on stretchable and wearable capacitive sensors are outlined in this review.

## 1. Introduction

With the recent development of flexible electronic materials, smart transducers, and wireless systems, wearable sensor technology has gained significant interest in the realization of personalized medical care [1]. The human body is considered a combination of chemical and electrical systems reinforced by a mechanical building; therefore, measuring such electrical activity allows for an objective assessment of health conditions. Miniaturized medical instruments, such as portable electrocardiography (ECG), glucometer, electronic sphygmomanometer, and paper-based diagnostics, have now been developed to offer low-cost, reliable, and easy disease diagnosis and monitoring at home. These instruments can provide early detection of chronic diseases, continuous health monitoring or recording for the long-term physiological response, and thus reduce the associated burden on the hospitals and the clinics. Wearable sensors can monitor human body conditions through muscles and body movements [2,3,4], vital signs (pulse, heart rate, temperature, humidity, blood pressure) [5,6,7,8], facial expression [9], vocalization [10,11,12], gas exposure [13,14], metabolism [15,16,17], and electrophysiological signals [18,19,20,21,22,23].

Wearable sensors for long-term electrophysiological signal monitoring, such as ECG (Electrocardiogram), EMG (Electromyogram), EEG (Electroencephalogram), EOG (Electrooculogram), and ECoG (Electrocorticogram), have been widely used to obtain physical and cognitive functions as the basis for health monitoring and diagnosis. In these devices, the electrodes act as the main sensing element for detecting the signal from the human body. Conventional wet Ag/AgCl electrodes are used to record the electrophysiological signals for short-term clinical use; however, they are not suitable for long-term applications in wearable technology because the soft electrolytic gel used between the electrodes and the skin usually dries out with time. The gel used in the electrodes is designed to enhance the adhesion and minimize the impedance at the electrode–skin interface. The utilization of Ag/AgCl electrodes can lead to skin irritation and allergies [24,25] and achieve skin contact through adhesive tapes [18]. This contact often results in signal noise from motion artifacts, owing to relative sensor slippage at the interface of electrode and skin [26]. In addition, these have limited options in mounting at the location of the skin and are uncomfortable for long-term usage because of their bulkiness and thickness [27,28]. Still, these are being utilized as the standard medical ECG sensors for long-term monitoring [29]. Ionic gels [30,31,32] or hydrogels [33,34,35,36] have emerged as one of the most promising options for long-term cutaneous electrodes due to their better stability. In addition, they can also be easily integrated with the fabricated sensor devices [30]. In contrast, dry electrodes are not well-adaptable to the curvilinear surfaces of skin [37,38], and the signal/response shows large motion artifacts. The utilization of microstructures for dry electrodes reduces electrode impedance and enhances the surface area [39,40], but motion artifacts are likely to appear during recording due to the absence of gel. In this context, several flexible/stretchable surface (non-invasive) dry electrodes with large deformability and good softness to maintain intimate conformal skin contact have already been introduced for long-term monitoring of electrophysiological signals [41,42,43,44,45,46,47,48]. Recently, the utilization of conducting polymers in dry electrodes for flexible/stretchable electronics has been shown to be beneficial for long-term monitoring [30,49,50]. Meanwhile, epidermal electronic systems (EESs) with lightweight, low-modulus, and ultra-thin form factors have also played an important role in overcoming the problems raised by rigid dry electrodes [19,44,51,52,53,54,55].

The design and development of flexible/stretchable dry electrodes with good adherence to biological tissues is in great demand due to the complex attributes of the human body. However, the major difficulty is finding the appropriate materials with good flexibility and conductivity, although some other relevant features such as bio-compatibility, durability, weight, size, etc. [56,57], are also important for long-term use. In this context, metals are good conductors; however, they are difficult to compress or stress and are not flexible. In contrast, traditional flexible materials provide low conductivity due to the poor density of the charge carriers. To address these shortcomings, two different strategies have been introduced: (i) fabricating thin conducting layers on flexible substrates using various polymeric materials such as polyvinylidene difluoride-(trifluoroethylene (PVDF-TrFE) copolymer [58], polyethylene naphthalate (PEN) [59], parylene [60], polyimide (PI) [61], and polydimethylsiloxane (PDMS) [62]; (ii) designing flexible/stretchable interconnectors from specifically designed materials [63]. The electrodes with high conductivity and stretchability are reported in several studies [19,26,64,65,66]. Various printing processes and fabrication strategies [67,68,69,70,71] have also been developed to fabricate electrodes with good skin contact [72]. The electrodes of wearable sensors have been developed from various materials with good conductivity, such as carbon-based nanomaterials [73], including graphene [74,75], carbon nanotubes (CNTs) [76], carbon fibers [77], or metals such as gold [18,21,22,78] or metallic nanoparticles including nickel and silver [79]. Among them, CNTs have become promising candidates due to their good mechanical stability, excellent flexibility, and high conductivity [80,81]. Usually, thin films of CNT are employed as the sensing materials in wearable dry electrodes with high performance, in which random networks of CNTs are formed via solution-based processes using low temperature and ambient atmosphere [82,83,84]. Stretchability is a critical factor that affects stability, sensing precision, adhesion to the skin, and repeatability of skin mountable sensors. These parameters are measured as the capability of sensors with regards to following the motion of skin that causes the deformation of skin up to 30% [85]. Skin mountable sensors with a unique structure such as serpentine paths [86], self-similar designs [87,88], or fractal geometries [89,90] are developed by either intrinsically thin film or soft materials on elastomer substrates. These strategies could reduce the mechanical impedance mismatching of electrodes to the soft curvilinear tissues by promoting skin adhesion through van der Walls forces [72,91,92,93,94,95]. Pressure-sensitive adhesives could be utilized to provide reversible adherence for the long-term integration with skin [96,97].

**Figure 1:**
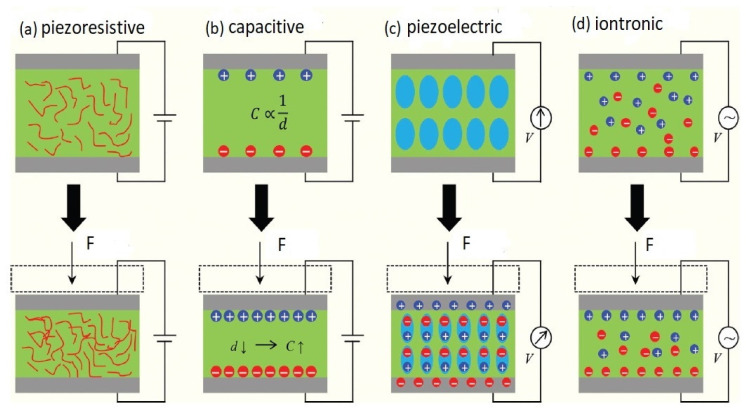
Schematic representation of four strategies used to detect or measure electrophysiological signals. Reproduced with permission from ref. [124]. Copyright 2018, Royal Society of Chemistry.

As shown in Figure 1, there are mainly four different categories of sensing strategies for measuring electrophysiological signals, viz. piezoresistive [98,99], piezoelectric [100,101,102,103], iontronic [104,105], and capacitive [106,107,108,109]. Among them, the capacitive sensors possess several advantages, such as reusability, sterilization, and reduced leakage currents (eliminating the electrical short circuits between electrodes and biological tissues) [110]. Capacitive coupling between electrodes and skin, using dielectric layers, allows the electrode materials to be encapsulated from the surrounding environment. It combines flat electrodes or conductive fabrics with different types of insulators, such as PDMS [111], polyimide varnish, cloth [112], and silicon dioxide [18]. These electrodes are affixed to the skin with tapes, belts, or caps to ensure intimate skin contact and avoid motion artifacts [25]. Deformable capacitive sensors have been designed to measure the strain or pressure on the skin [113,114]. Capacitive sensors also follow the principles of an epidermal electronic system (EES), with key determining factors, such as effective mechanical modulus, thickness, and area mass density [72]. Several studies [18,21,22,115,116], which used capacitive sensors for long-term monitoring, have achieved good signal-to-noise ratios (SNRs). Capacitive sensors also have great potential for disease identification using machine learning algorithms adopting strategies such as feature engineering [117,118] and deep learning [119,120,121,122,123].

In this review, we comprehensively focused on the design and application of wearable and flexible capacitive sensors for long-term health monitoring. Our study also included the sensing materials, sensing mechanisms, and fabrication strategies of wearable capacitive sensors. Finally, the potential applications of wearable capacitive sensors, along with some challenges, research gaps, opportunities required, and future research directions, have also been discussed.

## 2. Sensing Strategies

There are four main types of sensing strategies, as shown in Figure 1, which have been used to detect or measure electrophysiological signals, namely piezoresistive, capacitive, piezoelectric, and iontronic sensing.

### 2.1. Piezoresistive

The piezoresistive sensor is one of the sensors which are widely applied to read out the physiological information of human beings because of its simple design, easy readout, and facile operation [99,125]. Generally, such sensors demonstrate fast response and recovery speed [56] and hence, are widely used in flexible and wearable devices [126]. The principle of piezoresistive sensors is based on the changing of contact resistance (R_c_) between two materials by applying a force (F), as depicted in Figure 1a. This force is the key factor in changing the electrical signal within the contact region [127]. When force is applied to a device, the contact resistance changes following a power law, R_c_~F^−1/2^, resulting in a high sensitivity at low forces and a large operating range. In general, the relative change of strain sensitivity (Δ*R*/*R*) can be expressed as:

(1) $$\frac{\Delta R}{R}=\frac{\Delta \rho }{\rho }+\left(1+2\upsilon \right)\varepsilon =G_{\varepsilon }$$

where *ρ* is the resistivity of the sensing material, Δ*R* is the change of resistance, *R* is the unstrained resistance, *ν* is the Poisson’s ratio, *ε* is the applied strain, and *G_Ɛ_* is the gauge factor (GF), which indicates the measurement sensitivity determined by the variation of geometry and resistivity. In general, two methods can be used to increase the range of GF: (i) changing the contact region of the conductive elements and (ii) tunneling effect. The micro-scale cracks in thin conductive films provide a suitable media changing the contact region of films and exhibit a high gauge factor [99,128,129,130,131]. The crack-based piezoresistive sensors provide high sensitivity with small deformations. Amjadi et al. [132] demonstrated strong piezoresistivity of a nano-composite-based strain sensor, with an enhanced gauge factor using the tunneling effect and thus receiving high stretchability. Based on the literature, piezoresistivity sensors are used mainly as wearable pressure and strain sensors with applications in motion detection, pulse detection/monitoring, sound signal/vibration recognition, tactile sensing, human–machine interfacing, etc. As a result, they are generally employed in e-skin (Electronic-skin) [56,133]. Piezoresistive sensors have also been utilized to quantify temperature, pH, analyze concentration, humidity [134], and blood pressure [98]. Kong et al. [135] developed a skin-inspired hierarchical structure to handle the challenge of the mutually exclusive nature of two key parameters: a wide linear range and high sensitivity in e-skin at a time. They used reduced graphene oxide/poly (3,4-ethylene dioxythiophene): poly-(styrene sulfonate) aerogel to unify the contradiction by piezoresistive mechanism. The structure has shown the linear range of sensing up to 30 kPa without sacrificing the high sensitivity (137.7 kPa^−1^) of e-skin. Moreover, the structure also provided fast responsiveness (∼80 ms), low detection limit (1.1 Pa), and excellent stability and reproducibility (over 10,000 cycles), which are more crucial to detect small airflow, human pulse monitoring, and even sound-induced vibrations in e-skin.

### 2.2. Piezoelectric

Piezoelectricity is also a common transduction method in wearable electronics. Figure 1c depicts the sensing mechanism of a piezoelectric sensor, where an electrical polarization (localization of negative or positive charge) appears inside a material when a mechanical force is applied in a particular direction. This change of polarization results in a voltage difference on the opposite surfaces of the material. Some inorganic piezoelectric materials such as gallium nitride (GaN), poly(vinylidene fluoride-, lead zirconate titanate (PZT) [100,101], and zinc oxide (ZnO) nanowires [136] have been employed to develop flexible strain or pressure sensors in wearable applications by coating or imparting them into flexible polymers. Organic material poly(vinylidene fluoride-co-trifluoroethylene) PVDF-TrFE [102,137] is a particularly attractive material for piezoelectric sensors compared to others because of its good mechanical flexibility, cost-effectiveness, biocompatibility, chemical inertness, and good piezoelectric coefficient. The piezoelectric effect has been widely utilized for highly sensitive sensors in wearable electronics with a fast response time [138]. Applications of piezoelectric sensors in wearable electronics include measuring pulse waveform [101], body movement detection [100,137], dynamic pressure measurement in sound and mechanical vibration [56], small power consumption, and self-power sensing devices [136,139], and conducting the biomechanical devices [140]. Park et al. [141] have presented a real-time self-powered radial/carotid artery pulse monitoring system to overcome the shortcomings regarding the power consumption issues in wearable electronics using a piezoelectric sensing mechanism. In this study, high-quality ultrathin inorganic piezoelectric PZT film is transferred onto an ultrathin plastic of 4.8 µm thickness using inorganic-based laser lift-off (LLO) approach. They achieved good conformal contact to the complex texture of rugged skin with a sensitivity of ≈0.018 kPa^−1^, good mechanical stability, and a response time of ≈60 ms. The finding of a correlation between piezoelectric pulses and their respective blood pressure waves is always a challenging issue for accurate measurement of blood pressure. Yi et al. [142] have also explained the dynamic nature of arterial pulse and a continuous blood pressure measurement system addressing the issue that explored the feasibility of obtaining continuous blood pressure monitoring in wearable electronics with fewer motion artifacts. Muscle-fiber-inspired piezoelectric textile sensors with tunable mechanical properties have been developed by Su et al. [143] for physiological signals monitoring to fulfill the demand for highly stretchable, biocompatible, and robust features for next-generation biosensors in wearable electronics. Herein, polydopamine (PDA) is scattered into electrospun barium titanate/PVDF (BTO/PVDF) nanofibers to increase the mechanical strength, interfacial adhesion, and piezoelectric properties of the developed sensors. They demonstrated an outstanding sensitivity (3.95 VN^−1^). Furthermore, to test the long-term stability and robustness of their developed sensors, a linear motor at a frequency of 1 Hz was used to perform cyclic loading and release 5 N and observed the output voltage did not appear to drop by more than 3% after 7400 cycles, indicating excellent mechanical durability. This approach paves a cost-effective technique to develop high-performance self-powered bioelectronics sensors in wearable electronics for personalized healthcare.

### 2.3. Iontronic

Iontronic sensing is an advanced sensing mechanism introduced with a significant development to meet the challenges of device sensitivity and parasitic noises. An ionic-electronic interface of nano scale distance is formed between the electrolyte and electrode in iontronic sensors. Figure 1d shows the scheme for iontronic sensors. The operating mechanism of such sensors is based on the changing of the area between active material and electrode with the applied voltage. When a voltage is applied, the respective counter ions are attracted to the electrode interface, increasing the contact area and resulting in an ultra-high capacitance per unit area [144]. This capacitance of iontronic sensors is usually 1000 times more than a metal oxide’s parallel plate capacitor. Iontronic pressure sensors convert the applied force into a capacitance change under the change in the electrical double layer (EDL) at the interface of the electrode and the dielectric layer. Super capacitors based on the EDL mechanism have been largely utilized as energy storage devices based on the high surface area that provides a high energy density. Ionic gels with enormous negative and positive ions are entrapped spatially between both electrodes. As a result, a negative ion is attracted to the positive ion and forms two EDLs, as the applied voltage increases. This sensing mechanism of EDL with ionic gel obtains the pressure measurement by a package formed fully of soft materials [105]. The mechanism provides good responses to the body while minimizing the allergic effect on human skin and eliminating potential electric shocks to the human body; therefore, EDL integrating ionic materials such as ion gel, and ionic liquids, has recently been considered broadly for wearable sensing applications [104,105,145,146,147,148]. Applications of iontronic sensors in wearable electronics include measuring arterial pulse waveform [104], real-time health monitoring [105,148], and tactile sensation [104,106,145].

### 2.4. Capacitive

Recently, capacitive sensing has become a more attractive and popular sensing mechanism for mechanical stimuli, especially in the sense of touch, with good power consumption, sensitivity, and adaptive configurations of sensing [106,149,150,151,152,153,154,155,156]. Capacitive pressure sensors have been widely applied in consumer and industrial applications. Recently, their applications have been expanded to different pressure sensing interfaces for human beings, including electronic skin (e-skin) mimicking tactile sensation [114,154,157,158], body pressure mapping [149,151], and joint bending detection [79,149]. The enhancement of the flexibility of the electrode material is an important issue since it is the primary component of the wearable sensor [114,149,159]. Conductive nanomaterial [114,149] and polymers [113] have been used as electrode materials in capacitive sensors. Moreover, modified sensing interfaces and structures are also explored to further enhance the sensitivity of the sensor [113,152]. Bao and his colleagues have proposed a series of capacitive sensors for electrochemical sensing applications [114,153,154]. In this sensing technology, the parallel-plate technique is commonly employed for designing the mainstream capacitive sensor since it is comparatively easy to develop and construct a straightforward model. The change in capacitance is represented by the following equation:

(2) $$C=\varepsilon \frac{A}{d}$$

where *d* is the distance between the plates, *A* is the area of each plate, and *ε* is the permittivity of the dielectric material. Here *ε* is always constant while *A* and *d* are varied with the external forces, as depicted in Figure 1b. An applied force deflects the plates, and the distance of plates becomes shortened, resulting in the change of capacitance [124]. This change could be either linear or non-linear, and the capacitance is generally several picofarads (pF).

**Figure 2:**
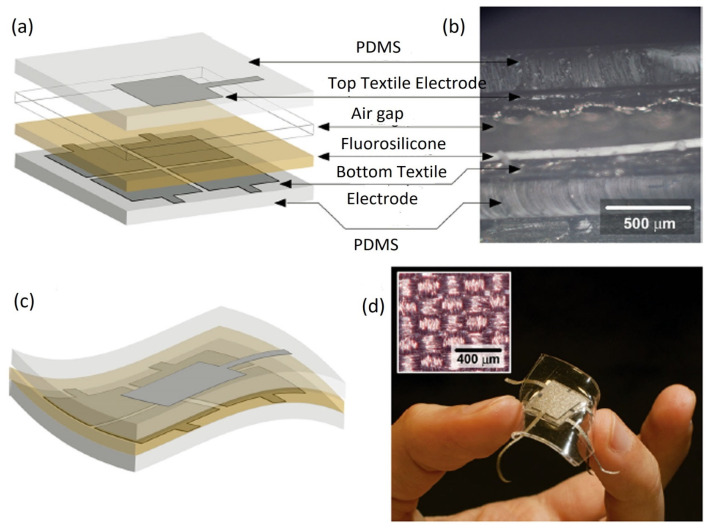
(**a**) A flexible capacitive textile sensor architecture. (**b**) Cross-sectional view of (**a**). (**c**) Illustration of a designed flexible capacitive sensor. (**d**) Optical microscopic exhibition of a conductive textile electrode. Reproduced with permission from ref. [166]. Copyright 2014, Wiley-VCH.

The wearable capacitive sensor can measure changes in capacitance because of various forces such as pressure, strain, and torsion [106]. The change in capacitance represents the variation of dimension or permittivity induced by physical, chemical, or biological stimuli. This sensing technology has mainly consisted of the substrate, electrode, and active materials sandwiched between two electrodes. They provide good sensitivity [160], possess better temperature tolerance than resistive sensors, and have sufficient frequency response [151]. In addition, the sensitivity could be optimized to alter ε various selection of dielectric materials such as elastomers [161], ionic solution [145], SiO_2_ [162], air gap [163], polyimide [164], and 3D fabrics [165]. The schematic representation of typical flexible parallel plate capacitive sensor architecture is illustrated in Figure 2. The key concept of a robust flexible (textile) capacitive sensor can be demonstrated by the top and bottom non-stretchable copper textile electrodes separated by a dielectric layer of floating fluorosilicone film with an intrinsic dielectric constant and good mechanical properties. Moreover, three-axial forces in the capacitive sensor are introduced to quantify and measure the flexibility, stability, robustness, and sensitivity with the aid of quadripartite electrode (textile) and air fluorosilicone dielectric [166]. The flexible capacitive sensors with the parallel plate have been implemented for monitoring the motion of the finger and wrist [107,167,168], the beating of the heart, and breath analysis [169,170]. Planar Interdigitated Capacitor (IDC) is an interesting configuration widely utilized in flexible capacitive sensors [108]. When the electric field passes through the dielectric layer upon approaching the object, the capacitance of the sensor is changed. This strategy is also utilized in touchpads with complex surfaces [108,109,171] and in stretchable insoles to detect gait [172].

In addition to the aforementioned categories, there are some other mechanisms to transduce the signal, including field-effect transistor (FET)-based sensors and inductor-capacitor-resonators based on RFID (Radio Frequency Identification) tags [173].

## 3. Functional Materials for Wearable Sensors

The flexible/stretchable wearable sensors consist of mainly two components: substrate and active element/electrode with the interconnectors. Although organic materials have good mechanical flexibility and stability, they suffer from poor electrical performance. In contrast, inorganic materials have good electrical performance and poor mechanical responses, associated with rigidity and brittleness; therefore, organic and inorganic materials provide a good solution for developing compact sensors with mechanical robustness and high sensing performance. Scaling down dimensions and advances in synthesizing composites may assist in developing the desired devices. The most employed materials and their applications in the substrate and active element or electrode are discussed in the following sub-sections.

### 3.1. Substrate Materials

Flexibility, stretchability, comfort level, and long-term reliability of a wearable capacitive sensor are directly associated with the substrate. The substrate selection is highly crucial for designing and fabricating the sensors [174]. Among the materials, PDMS is widely used in the laboratory due to its stretchability, commercial availability, biocompatibility, hydrophobicity, non-flammable nature, chemical inertness, and easy processing; therefore, PDMS is widely used in microfluidic devices, prostheses, and wearable sensors [175]. Various types of elastomers have also been used to fabricate wearable sensors. For example, polyurethane (PU) and acrylic elastomer are used as skin sensors, as they are softer compared to PDMS. The maximum stretchability of single-walled carbon nanotube (SWCNT)/silicone rubber composites with PDMS have been reported to be 300% [176]. PDMS and polyurethane acrylate (PUA) are photo curable and can be used to create a pattern through traditional photolithography processes [177] and 3D printing techniques [178]. Ecoflex^®^ rubbers are skin-safe and highly stretchable with low modulus in wearable applications [149,179]. Excellent printability, good transparency (>85%), and good creep resistance allow them to have appeared in the electrochemical sensors as the substrate film [16,180,181]. Polyimide (PI) is another popular substrate for wearable sensors. It has good creep resistance, high tensile strength, good flexibility, and good resistance to acids or alkalis [182]. PI films play an important role in the micro-manufacturing process with more diversity for designing as well as implementing wearable sensors. Apart from PI films, polymer fibers and textiles have also been employed to deposit various active materials as the core sensing materials in wearable electronics [64]. A summary of several widely used substrate materials in wearable electronics, including their pros, cons, and Young’s modulus, is depicted in Table 1. From the table, it is observed that Young’s modulus of Ecoflex is near to the magnitude of human skin epidermis and dermis, which indicates Ecoflex could be more adaptable to the human skin compared to other substrate materials in wearable electronics. 

### 3.2. Active Element/Electrode Materials

#### 3.2.1. Carbon Materials

Various types of carbon materials, including carbon nanotubes, graphene, and graphite, have been used to fabricate the capacitive wearable sensors as active or electrode materials. Among carbon materials, graphite with a 3D crystalline structure is softer, cleaves with low pressure, and has less specific gravity. It has recently been used for the development of pencil-on-paper of electronics [191,192]. There are two types of CNTs, namely SWCNTs and multi-walled carbon nanotubes (MWCNTs). Both types of CNTs were already employed to fabricate wearable sensors in flexible and stretchable electronics [193,194]. Previously, CNT powder was mixed with a polymer substrate to fabricate wearable biosensors, which have shown good mobility of ~10^5^ cm^2^V^−1^s^−1^ [195]. 2D carbon materials, such as graphene [196], have also been utilized for developing flexible/stretchable sensors due to their good mobility (2 × 10^5^ cm^2^V^−1^s^−1^) at room temperature, excellent thermal conductivity (5300 Wm^−1^K^−1^), and excellent mechanical properties (25% in-plane stretchability, high tensile strength (125 GPa), and high Young’s modulus (1 TPa)). Graphene has also been used to construct electrodes in capacitive sensors and as filler material in piezoresistive composite sensors, such as CNTs. Furthermore, both CNTs and graphene have been used to construct fully transparent sensors due to their optical transparency and high flexibility as well as softness [197,198]. These materials are particularly suitable for developing high-performance devices, such as top-gated transistors [199,200,201]. Some conventional materials are also utilized to synthesize carbon materials due to their being low-cost and environmentally friendly. For example, the PI film can be directly scribed by laser to produce functional patterns on porous graphene employed in acoustic sources and artificial throat detection [12,202]. Due to their promising characteristics, carbon materials have been widely used as active materials or electrodes in flexible and stretchable wearable biosensors and promising active materials.

#### 3.2.2. Metallic Materials

Metals are largely utilized to construct wearable sensors due to their excellent conductivity. They are usually found in the form of (i) nanowires (NWs) or nanoparticles (NPs); (ii) configuration in flexible/stretchable structure; (iii) liquid state at normal temperature. NWs and NPs are the most attractive active materials to fabricate the composites of piezoresistive and conductive ink as the fillers in sensors, whereas silicon NWs [203], metal NWs [204,205,206], transition metal dichalcogenides (TMDCs) [207], and silver NWs (AgNWs) are employed onto PDMS to develop resistive sensors [132,208]. Conductive inks with metal NPs have been cast and annealed to construct capacitive sensing electrodes on the substrate surface. Rogers et al. [209] and Sekitani et al. [210] have reported the structures of stretchable metals for developing stretchable electronic devices with innovative configurations. Strain sensors [211,212,213], soft wire [214], pressure sensor [215], and antenna [216] were constructed as microfluidic devices by injecting liquid metals into the channels. Fabricated devices with liquid metals were able to resist the deformation in micro channels at high strain up to 800% [217].

Hard metals and semiconductors can also act as active materials. Solid hard metals, such as Au, Al, Cu, Ti, Cr, and Pt, are intrinsically conductive; however, they become flexible when prepared in the form of thin films. These thin metallic films are widely used to develop electrodes, contact pads, interconnect, and circuit components, such as a resistor, capacitor, and inductor. The fracture strain of these metals is less than 1% due to their ductile nature; however, the stretchability of these metallic films may be enhanced by more than 100% when they are designed into specific structures, such as pre-strained bulking [218], fractal [219], self-similar serpentine [19], and helical [220].

Apart from the metals, the active components in diodes and transistors are also made up of some inorganic semiconducting materials, such as silicon [221], ZnO [222], GaN [223], GaAs [224], InP [225], and organic semiconductor materials, such as poly (2,5-bis (3-hexadecylthiophen-2-yl) thieno [3,2-b] thiophene) (pBTTT)) [226], poly(p-phenylene)vinylene [227], and poly(3-hexylthiophene) (P3HT) [223]. These semiconductors are usually patterned into nanowires [225], nanoribbons [228], and nanomembranes [229] by complementary metal-oxide-semiconductor (CMOS) processes. Some organic polymers such as poly-(3,4-ethylenedioxythiophene) (PEDOT) polymer is particularly attractive in wearable sensors as the active element due to their high transparency, good thermal stability, flexibility/stretchability, and tunable conductivity. PEDOT: PSS (polystyrene sufonate) has been commercialized as a conductive polymer because of its excellent solubility in water, which made it more compatible with some conventional processing techniques, such as spin-coating and inkjet printing, dip-coating, etc. [230]. Unfortunately, the PEDOT: PSS film cannot be bent continuously or stretched because of its intrinsic hardness. Such bending or stretching can lead to fracture and reduction in the film conductivity; however, PEDOT: PSS ink can be easily printed and entered into porous substrates, such as cellulose paper [64]. Other polymers, such as PVDF-TrFE, polypyrrole (PPy), poly aniline (PANI), and polyacetylene (PA), are also used for developing wearable biosensors [231]. Park and co-workers have introduced a conductive polymer composite that led to high conductivity (σ~2200 Scm^−1^), even with a large deformation (100% strain) through the rubber fibers of electrospun poly (styrene-block-butadiene-blocks-styrene) (SBS) embedded with the silver nanoparticles (Ag NPs) [232]. Similarly, Shang et al. [233] have fabricated an elastic composite of conductive nanocomposites made from MWCNTs and polyurethane (PU) with stretchability greater than 100% and an initial conductivity greater than 5.3 Scm^−1^. These studies suggest that composites of conductive polymers and fillers can be used to fabricate wearable sensing devices with improved sensing properties. A summary of several widely used flexible electrode materials in real-life applications is depicted in Table 2, including their advantages, disadvantages, applications, electrical property, and Young’s modulus.

### 3.3. Hydrogels/Ion Gels in Wearable Electronics

Recently, ion/ionic gels and hydrogel-based electrophysiological sensors as wearable devices have attained great attention in soft electronics for long-term signal monitoring and recording. Soft and stretchable devices are an emerging field. Hydrogels/iongels are compelling materials because of their softness, biocompatibility, chemically tunable, and ionically conductive properties. These types of sensors interface iongels/hydrogels with rigid metallic electrodes to human skin or electronic circuitry. Metals have good electrical properties, but their large Young’s modulus (~GPa) is mismatched mechanically to the human skin. On the contrary, these gels have moduli from Pa-MPa, which is similar to the moduli of human skin (epidermis 140–600 kPa, dermis 2–80 kPa [72]). As a result, wearable electrophysiological sensors having these gels can easily adjust to the human skin during deformation due to body movements and be compliant.

#### 3.3.1. Hydrogels

Hydrogels are soft materials with elastic nature, including a three-dimensional polymer network [245]. These are widely used in the skin mountable electrophysiological sensors demonstrating promising devices as transparent and stretchable electrodes. Shay et al. [33] developed a soft and deformable ECG electrode combining a liquid metal (EGaIn-eutectic gallium indium) and hydrogel, which provides low impedance at relevant low frequencies (1–50 Hz) and better signal-to-noise ratio compared to commercial ECG electrodes. Interestingly, it has the advantage of reusability and the softness of hydrogel could be modified without compromising the electrical behavior of electrodes. In [36], an Au film-based electrode with a conducting polymer (PEDOT) is tightly bonded to a double-network hydrogel to measure electrophysiological signals (EMGs), which are shaped and conformable to the human skin. They showed that the developed electrode has a stable resistance (35 ± 5 Ω sq^−1^), even with a successive stretching of 20%, double-layer capacitance (9.5 ± 0.3 mF cm^−2^) at the interface of composite layers against external noises, and a stable impedance at the frequency of 5–500 Hz which is the typical range of EMG signals. Hydrogel-based sensors usually have low inferior anti-freezing and strain sensitivity properties, which limit the usage of these sensors in wearable electronics. Consequently, developing an antifreeze hydrogel sensor with a high tensile, quick repair, and fatigue resistance remain a challenge. Wang et al. [246] presented a stretchable, self-wrinkled, biocompatible, and anti-freezing hydrogel-based sensor with PEDOT: sulfonated-lignin as the conducting materials on a poly acrylic acid (PAA) for wearable applications. They demonstrated that the developed electrode provides superior GF of up to 7 with a strain of 100% and good anti-freezing properties. For the application of heart rate monitoring on sleeping conditions with wearable capacitive ECG sensors, Feng et al. [35] proposed hydrogel-based conductive textile electrodes to obtain good quality signals to overcome the usual challenges such as slow coupling capacitance, composed of bed sheet, human skin, sensing electrodes, and pajamas, mainly caused due to the low relative dielectric constant between pajamas and bed sheet. In this work, the hydrogel layer is applied as an array pattern onto textile to be a sensitive electrode to enhance the coupling capacitance and lower impedance which are more crucial to improve the quality of raw signals. Currently, conductive hydrogels (CHs) are widely used to develop soft electrodes incorporating conductive polymers, metal-based nanowires, and carbon materials, but these approaches are costly. Moreover, conductive materials tend to aggregate with the hydrogels, which highly affects conductivity. Most importantly, the damaging reasons of conductive materials to the human tissues are still unknown. So, some researchers tried to resolve these challenging issues of CHs by developing mussel-inspired mechanisms. For instance, Pan et al. [34] presented mussel-inspired hydrogel electrodes with nanocomposite to detect electrophysiological signals (ECG and EMG) from the human body, which have reusable, adhesive, editable, conductive, and injectable properties. Carbon nanotubes (SWCNTs) could also play a great role in resolving such categories of challenges because of their exceptional thermal, mechanical, and electrical properties. Two different approaches were examined by Gilshteyn et al. [247], a simple SWCNT film transfer onto prepared hydrogel and film deposition onto pre-stretched hydrogel. From both methods, it was observed that the developed hydrogel-based electrodes with SWCNT are stretchable, sticky, intrinsically soft, highly transparent, electrically conductive, and well conform to human skin. Beyond the applications in wearable electrophysiological sensors, hydrogels are widely studied and recently applied in drug delivery, skin dressing, tissue repairing, cell culture, sewage treatment, triboelectric nanogenerators, bioelectronics, microfluidics, soft robotics and actuators, electronic skin (e-skin), etc.

#### 3.3.2. Iongels

Stretchable/flexible electronics are twistable, mechanically bendable, foldable, and can easily conform to non-planar surfaces such as human skin. Iongels have good mechanical conformality, biocompatibility, transparency, and stretchability. So, recently Iongels have been of considerable attention in wearable electronics. It indicates a novel category of stretchable materials composed of electrolyte solutions and polymer networks where the ionic liquid is immobilized in the polymer matrix [31]. Based on the category of solvents in the polymer matrix, iongels could be classified into non-aqueous and aqueous. Ag/AgCl electrodes require an electrolyte to control the decrease in impedance at the interface of electrode and skin during signal recording in long-term, but the electrolyte dries out after several hours when it is exposed to air and increases the impedance at the interface and resulting in the signal quality is reduced. Moreover, refilling electrolytes is time-consuming and a hassle, and patients also feel discomfort and irritation with electrolytes in long-term monitoring. In contrast, the dry electrode is not well-adaptable to the curvilinear surfaces of human skin during body motion and results in large motion artifacts. Gel-based electrodes are a good solution to resolve these shortcomings of gold standard commercially available Ag/AgCl and currently developed dry electrodes for long-term recordings. A gel-assisted electrode with ionic liquid developed for long-term EEG recordings from the human body by Leleux et al. [30] incorporates iongels onto electrodes, consisting of Au and a conducting polymer (PEDOT: PSS). It observed that iongels-based electrodes provide better performance and low impedance over a long period at electrodes and human skin interface compared to Ag/AgCl and dry electrodes. Considering the toxicology issue of ionic liquids (ILs), Isik et al. [32] developed cholinium-based ion gels for long-term electrophysiology recordings from the human body where their prepared ion gels were incorporated onto the electrodes, made of Au and PEDOT:PSS. The gel is composed of cholinium lactate IL and photopolymerization of poly(cholinium lactate methacrylate) network and observed good performance (rheological and electrical properties, good thermal stability, low toxicity, good ionic conductivity of ion gels, low impedance at the interface of electrode and skin) for the various composition of IL and polymer with different temperatures compared to Ag/AgCl electrodes. Cholinium ionic liquids and ion gels are highly appealing for long-term cutaneous electrophysiology and other biomedical applications due to their low toxicity and superior ambient stability. Recently, an organic electrochemical transistor (OECT) with ion gel in its gate was widely used to record high-quality bio-potential signals from the human body because of its key advantages such as biocompatibility, high trans-conductance, and low operating voltage [248]. The transistors with a direct electrolyte gate limit their operation to collect signals in the long term due to the short time existence of an electrolyte. Moreover, the ionic-gated transistors (IGTs) have good mechanical, chemical, and physical stability [249]. Printing technologies and additive manufacturing have received tremendous attention nowadays as the versatile platform for the on-demand fabrication of devices and objects with their excellent functionality and control characteristics. In this context, ion gels with 3D printability play a great role in the next-generation bioelectronics devices. In [250], the authors presented biocompatible, thermoreversible (85–110 °C), and 3D printable ion gels for biomedical applications, which are processed by direct ink writing, and ion gels are prepared with taking the advantages of polyvinyl alcohol/phenol interactions to the biocompatible cholinium carboxylate ILs to gelify. The achieved ion gels were soft, stable, good flexible (ionic conductivity of 1.8 × 10^−2^ S cm^−1^, Young’s modulus of 14 to 70 kPa). Aguzin et al. [251] also prepared ion gels for body sensors and bio-electrodes, considering the lack of biocompatibility of conventional ILs and polymer matrices where tannic acid is used as the cross-linker in the gelatin matrix and three different biocompatible cholinium carboxylate ILs. Their prepared ion gels provided good ionic conductivity (0.003 to 0.015 S cm^−1^) and were flexible and elastic with Young’s modulus of 11.3 to 28.9 kPa at room temperature, which is more adaptable to human skin. Ion gels are also greatly employed in energy storage devices (fuel cells, batteries, supercapacitors), e-skin (electric double layer transistors, strain sensors, pressure sensors, etc.), soft actuators/robotics, flexible displays, transparent loudspeakers, underwater microphones, electroluminescent devices, drug delivery systems, biochemical and electrochemical sensors, gas separation, field-effect transistors, etc.

## 4. Fabrication of Wearable Sensors

**Figure 3:**
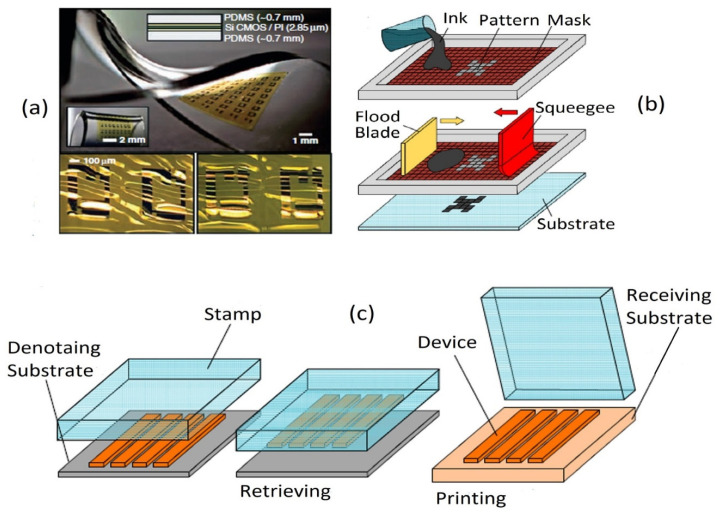
(**a**) Fabricated stretchable IC (integrated circuit) based on CMOS technology. Reproduced with permission from Ref. [279]. Copyright 2008, American Association for the Advancement of Science. (**b**) A schematic diagram of the screen-printing process. (**c**) A schematic of the transfer printing process. (**b**,**c**) Reproduced with permission from ref. [254]. Copyright 2017, MDPI.

The fabrication strategies of a wearable electrophysiological capacitive sensor can be categorized into two main groups: (i) pattern transferring and (ii) compositing materials. Pattern transferring strategies; include lithography, micro-scale modeling, and various types of printing, such as inkjet printing, 3D printing, screen printing, and handwriting techniques. In this context, Rogers and co-workers have introduced a series of flexible as well as stretchable sensors with good flexibility and elasticity using lithography techniques to realize various ingenious geometries such as systems with strain, temperature, and electrophysiological sensors matched to the epidermis of human beings [72], feedback control systems with integrated electronic [28], thermal characterization with 3ω sensors [7], near-field communication with the epidermal coil, etc. The major concern of micro-scale fabrication is to overcome the adhesion between the mold and the processed materials; therefore, sophisticated geometric design and essential pre-treatment are needed to achieve the complete peeling-off process. Among the printing techniques, screen printing provides high throughput and low-cost processes for developing skin motion sensors. This technique is largely used to construct the electrodes and sensing elements for electrochemical and mechanical sensors [252,253,254]. A typical example is illustrated in Figure 3b. Herein, ink is deposited onto the surface of the substrate by vacuum filtration [128,255], Mayer rod coating [256] or spin/spray coating [169,170,257,258]. The major shortcomings of these techniques are the proper selection of ink materials, the low printing resolution (>10 µm), and slow processing time owing to solvent evaporation. Inkjet printing provides an accurate and rapid method for forming films; however, controlling the viscosity, surface tension, and solubility of the ink droplets onto the substrate via a nozzle or other compatible equipment is challenging. For example, Zheng et al. [259,260] developed a series of liquid metal printers to achieve simple and fast manufacturing of personal electronic circuits. Passive components, such as inductors [261], capacitors [262,263,264], resistors [265,266], and active components, such as LEDs [267,268] and thin-film transistors [269], are typically fabricated using this technique. In addition, this method is also utilized in wet etching to develop the subtractive pattern for making organic devices [270]. Three-dimensional printing is highly popular for building flexible/stretchable sensors [271,272,273]. The sensors developed using this approach can detect and distinguish human motions due to their high sensitivity and small size. Inspired by daily handwriting, innovative and ubiquitously available pens could be used to write conductive patterns on various substrates to fabricate the do-it-yourself, solvent-free, rapid, simple, and low-cost wearable sensors [271,274]. A high perfection strain sensor tattoo has been employed as a human–machine interface (HMI) device to control a robot arm with the help of AuNWs/PANI ink and the Chinese brush pen [275]. In addition, liquid metals are another important application of this approach. Liu’s group has previously introduced gallium-based geometries in diverse forms onto different substrates such as tape, paper, human skin, and wood [107,276,277,278].

Researchers are searching for a hybrid process for achieving multipurpose wearable sensors that can overcome the shortcomings of lithography and printing techniques. Recently, Mohan et al. [280] fabricated a series of high-performance stretchable sensors with “island–bridge” structures, where lithography was utilized to fabricate the serpentine interconnects, and screen printing was employed to construct the sensitive “island”. The laser scribing (LS) approach is another technique for manufacturing wearable sensors with high performance [12,189]. Direct laser writing (DLW) is largely utilized in micro-super capacitors with planar inter digitated electrodes [202], flexible strain or tactile sensors [281], micro ball lenses [282], 3D conductive carbon circuits [283], wireless pressure sensors [284], gas sensor [285], etc., due to its favorable hydrophilic wettability, high porosity, and good electro-conductivity. A typical fabricated stretchable CMOS with transfer printing is illustrated in Figure 3a. The target devices are fabricated by the contributing substrate. After that, it is transferred onto the receiving substrate with a viscoelastic stamp (usually PDMS), as seen in Figure 3c. The transfer printing technique has been successfully employed to fabricate some devices, such as the sensing elements in integrated circuits [286], light-emitting diodes [287], transistors [288], and so on.

## 5. Wearable Capacitive Electrophysiological Sensors

### 5.1. Electrode-Skin Model

**Figure 4:**
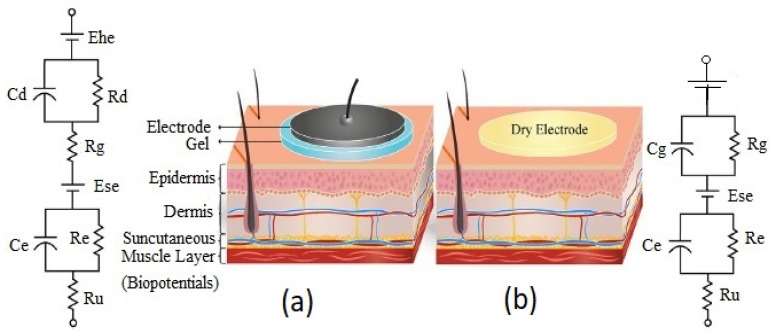
Schematics and corresponding electrode–skin interface models. (**a**) Pre-gelled wet electrode. (**b**) Dry surface electrode. Reproduced with permission from ref. [22]. Copyright 2019, Elsevier.

The main element of a bio-potential sensing device is the electrode which can be polarizable or non-polarizable, referring to what happens when current flows between electrode and electrolyte [289]. When current is applied to a perfectly polarizable electrode, no actual current passes through the electrode–electrolyte interface, whereas current flows freely in a perfectly non-polarizable electrode. Displacement current has, of course, appeared in polarizable electrodes. An additional interface is taken into account when bio-potentials are measured from the skin’s surface to mitigate the effects of the impedance between the electrode and the skin. The efficacy of the charge transfer from the body to the electrode is often assessed by measuring the impedance of electrode–skin contact [290]. The equivalent electrode–skin interface model of a pre-gelled wet electrode is illustrated in Figure 4a. Herein, a half-cell potential denoted by E_hc_ is developed at the interface of the electrochemical electrode-electrolyte. A resistor R_d_, leakage resistance (<1 k ohms) between the two layers, and a capacitor C_d_ are placed in parallel to develop a double-layer structure model at the interface. R_g_ indicates the resistance of ionically conducting gel. Dermis, epidermis, and subcutaneous tissues are the elementary layers of the human skin. The resistor, R_u_ denotes the dermis layer resistance, whereas the epidermis exhibits resistance, Re (<10 k ohms for wet gel and 10–100 k ohms for solid gel) and capacitance, C_e_ (50 nF for wet gel and 10–50 nF for solid gel), which are placed in parallel to develop electrode–skin interface model [22,291,292]. Dead cells make up the stratum corneum, the epidermis’ outermost layer. It is very dynamic and serves as an electrically insulating barrier to protect the deeper layers. Due to the ions’ semi-permeation through the stratum corneum, the difference in ion concentration produces the potential, Ese [289].

The signal of the dry electrodes is found to eliminate the deterioration due to the absence of gel as well as concerns regarding the skin dermatological responses, as shown in Figure 4b. These dry electrodes are divided into non-contact and contact electrodes, which can be further categorized into surface electrodes, penetrating electrodes, and capacitive electrodes based on the coupling between the skin and the electrode. In surface electrodes, the coupling between the skin and the electrode is capacitive because of the absence of conductive gel. This leads to unavoidable air gaps or bubbles between the skin and electrode that can act as the dielectric layer. Such air gaps or bubbles are observed when the electrodes are rigid and not adaptable to the skin surface [293]. The humidity of skin and sweat produced underneath the electrode generates a resistance between the skin and the electrode; therefore, resistor R_g_ and capacitor C_g_ are placed parallel to develop the electrode–skin interface model, as depicted in Figure 4b. Herein, a half-cell potential (E_hc_) is added to express the interface, Re is between 30 k ohms-1 M ohms, and Ce is 10–50 nF. The impedance of the dry electrode surface–skin interface is greater than that of the wet electrode. The contact impedance decreases with the application of pressure [45,293,294,295]. High hydration level or skin humidity reduces the skin impedance to enhance the skin dielectric constant and conductivity [296]. Usually, the electrode–skin impedance tends to decrease gradually after employing the electrodes on the skin due to the development of perspiration [297]; therefore, motion artifacts are initially high but decline gradually and attain a lower level than pre-gelled electrodes after a certain time [24]. Flexible/stretchable electrodes may provide possible solutions to handle the motion artifacts compared to rigid surface electrodes made using stiff materials [39,294]. There are several ways to develop flexible/stretchable surface electrodes, such as deposition of the thin metal film onto the flexible substrate [298,299], patterning the serpentine metal mesh onto thin silicone to develop tattoo-like electrodes [72], and fabricating textile-integrated electrodes [300] via metal electroplating [301], incorporation of conductive fillers into polymeric matrices to form polymer composites [302,303], and weaving or knitting conductive yarns into fabrics [304]. These flexible electrodes have better adaptability with the skin topology, hairs, and curvature than rigid ones; therefore, they can easily obtain lower impedance with a stable interface and provide better comfort to the human skin [298].

For penetrating dry electrodes, the electrodes are pierced into the skin surface by minimally invasive procedures, and the impacts of insulating and volatile stratum corneum are alleviated effectively [291]. The equivalent electrical model illustrated in [292,305,306]. A half-cell potential, E_hc_ added to the model to express the interface potential between the electrode and conductive layers of the epidermis. A capacitor, C_e_, and a resistor R_e_, are placed in parallel to develop a double-layer structure in the model. The resistance, R_u_ is only present due to the coupling between the electrode and conductive layers. Barbed micro-tip arrays [305], micro-machined sharp spikes [306], and hollow micro-needle arrays [307] have been designed and developed to penetrate the surface of the skin to measure electrophysiological signals; however, for long-term applications, they need to be further improved in terms of electrical conductivity, mechanical stability, and biocompatibility with skin.

In the capacitive electrode, the skin is isolated from the electrodes by an insulating layer or jointed with air, cloths, or various dielectric materials. Herein, a resistor R_i_ and a capacitor C_i_ contributed from the air or cloths or other dielectric materials such as PI, polymer fibers, and textiles are placed in parallel to develop the electrode–skin interface model, as illustrated in [22,25]. The interface could be expressed by a series connection of dielectric layer capacitors with a parallel connection of epidermis resistance, Re (100 kΩ–1 MΩ), and capacitance, Ce (10–50 nF). In most cases, the dielectric layer capacitance (1 pF–10 nF) dominates the interface impedance. Capacitive electrodes are safe for skin, easy to reuse and clean, electrically safe, and easily integrated with dielectric materials [18,21,22]. Capacitive electrodes are usually bent with the body movement, and unstable moving electric charges appear close to the electrodes. Artifacts due to the body movement for the capacitive electrodes are higher initially compared to pre-gelled wet electrodes, but gradually decline over time to a level less than that of wet electrodes; however, considering the proper shielding and buffering, size, and geometry of electrodes, the capacitive electrodes could perform similarly to pre-gelled wet electrodes in the clinical setting by considering factors, such as motion artifacts, and interference resistance from moving charges, and contact impedance [24].

### 5.2. Design

The stretchability of flexible skin sensors can be achieved at material and structural levels with less reaction force. The bending strain of materials decreases linearly with the increase in thickness from 100 nm to a few µm. For example, silicon nano-scale ribbons provide only a strain of 0.0005% with a thickness of 100 nm for a radius of curvature of 1 cm, while the strain is 0.1% with a thickness of 20 µm at the same curvature, which is below the fracture limits of 1%. It is possible to bend materials to obtain a strain of ~0.1% for a radius curvature of ~150 µm [209]. To create intrinsically rigid but stretchable materials on elastomeric substrates, two different approaches have been developed. The first approach follows the out-of-plane buckling of materials with the ultrathin (nano) scale to relieve the stress developed in the substrates. The second approach follows the stretchable interconnects as the bridges join together rigid islands. These islands usually take on functional components, such as commercial off-the-shelf components [308], electronics [36,88,218], and sensors [219,296]. Both approaches are widely utilized in the development of stretchable skin sensors.

#### 5.2.1. Out-of-Plane Design

**Figure 5:**
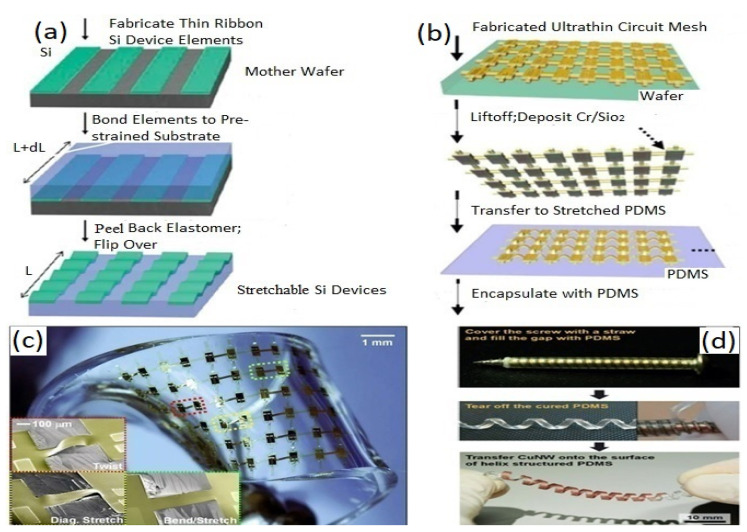
(**a**) Out-of-plane nanoribbons formation (Out-of-plane stretchable structure mechanism). Reproduced with permission from Ref. [317] Copyright 2006, American Association for the Advancement of Science. (**b**) Fabrication process to develop an array of CMOS inverters with stretchability at high levels through the non-coplanar mesh design strategy. (**c**) Optical image of a deformed stretchable array of inverters of CMOS, showing three classes of deformation: bending, twisting, and diagonal stretching (example of an out-of-plane island–bridge structure with high stretchability). (**b**,**c**) Reproduced with permission from Ref. [218] Copyright 2008, National Academy of Sciences. (**d**) A helical structured nanowire of copper (Cu NW)-based electrodes (Example of an out-of-plane structure with high stretchability). Reproduced with permission from ref. [316] Copyright 2014, Springer Nature.

The formation of an out-of-plane design with the buckling structure is shown in Figure 5a. Bonding of the nanoribbons onto the pre-strained elastomeric substrate and ultrathin ribbons can be achieved through a conventional lithography process. Releasing the pre-strain leads to wavy structures of periodic nature on both the ribbons and the substrate. The magnitude, *A*, as well as the wavelength, *λ*, of the wavy structures can be measured using the following equations:

(3) $$\lambda =2\pi h_{f}{\left(\frac{E_{f}}{3E_{S}}\right)}^{1/3}$$

(4) $$A=h_{f}\sqrt{\frac{\varepsilon_{pre}-\varepsilon_{applied}}{\varepsilon_{c}}-1}$$

where *h_f_* is the thickness of stiff ribbons, *E_f_* is Young’s modulus of the elastic substrate, *ε_applied_* is the applied strain, *ε_pre_* is the pre-strain level, and *ε_c_* is the critical strain for buckled ribbons. The peak strain, *ε_peak_* in the ribbon is approximated by the equation:

(5) $$\varepsilon_{peak}\approx 2\sqrt{(\varepsilon_{pre}-\varepsilon_{applied})\varepsilon_{c}}$$

So, the maximum stretchability of the wavy structures could be measured by *ε_peak_*.

The recent improvement of wavy design has led to more complex 3D structures with buckles at higher orders to form miniaturized stretchable or flexible electronics with complicated spatial distribution [309]. The stretchability of a wavy design is measured through the pre-strain levels of the substrates and materials. The bending curvature can also be determined using Equations (4) and (5), in applications requiring larger stretchability and less complex fabrication procedures. The wavy design has been widely utilized to integrate silicon [310], graphene [311], carbon nanotubes [312], and ferroelectrics [313] in various forms, including nanowires [314], nanoribbons [310], and nanomembranes [315]. The stretchability of island–bridge configurations is obtained either by the planar deformation of interconnects [89] or by the deformation of spatially buckled bridges [218]. Figure 5b illustrates the fabrication process to achieve the stretchability of mesh design strategy with the elastomeric substrate integration to develop an array of CMOS inverters. Figure 5c displays the optical image of a stretchable array of inverters of CMOS. Novel spatial helical structures were introduced by utilizing the diverse twisting modes to be employed as power harvesters as well as sensors. A helical spring providing high stretchability based on copper nanowires [316] is illustrated in Figure 5d. This structure can be implemented in various wearable electrophysiological sensors to easily achieve deformation.

#### 5.2.2. In-Plane Design

**Figure 6:**
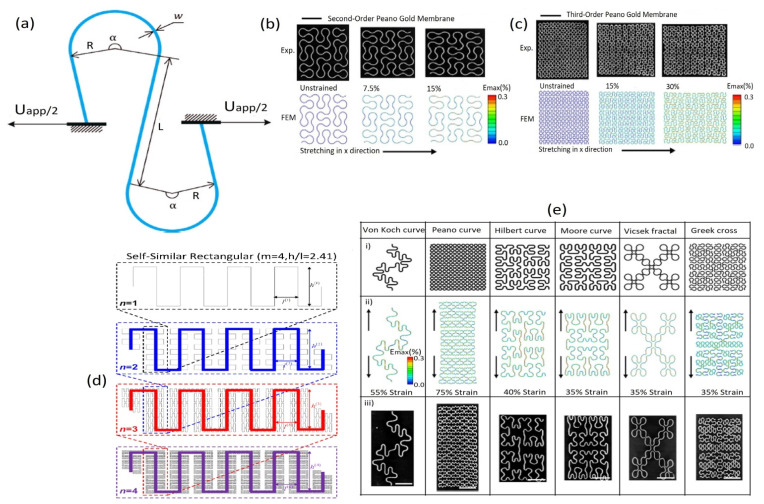
Fractal and serpentine structure mechanisms: (**a**) A serpentine interconnect is subjected to the axial stretching (Uapp) at the endpoints. Reproduced with permission from ref. [321] Copyright 2016, Elsevier. (**b**) Differences between the experimental optical images and FEM for the second-order structures with the stretching at different levels. (**c**) Differences between the optical images and FEM for the third-order structures with stretching at different levels. (**d**) Schematic illustration of self-similar serpentine interconnects with the geometric construction. Reproduced with permission from ref. [323] Copyright 2013, Elsevier. (**e**) Representation of deformation of various fractal structures [89]. (**b**,**c**,**e**) Reproduced with permission from ref. [89] Copyright 2014, Nature Publishing Group.

Improved sensor designs without using pre-strained substrates can be obtained with the in-plane island–bridge configurations bonded onto elastomeric substrates in the form of fractal or serpentine [89,318,319,320] structures. Sometimes, island–bridge structures are fabricated with the continuous self-similar fractal or serpentine structures in the sensors as well as interconnect. Fan and colleagues [321] introduced an analytic model which depends on the finite deformation theory for in-plane serpentine interconnects. A serpentine interconnect is facilitated by the three straight wires, where the length *L* or *L*/2 is linked to two arcs with the arc angle α and identical radius *R*, illustrated in Figure 6a. Then ŵ = w/*R* (width/radius), arc angle, α, and *Ĺ* = *L/R* (arm length/radius) are the three dimensionless parameters used to express the shape of the serpentine interconnect. If ŵ is smaller than 0.5, then interconnect is non-buckled, and such interconnect is modeled as a curve similar to the Euler–Bernoulli beam. If, at the ending, the serpentine interconnect is bonded to the tensile displacement (*U_app_*/2), and then the effectively applied strain, *ε_app_* of the serpentine interconnect is represented by the equation:

(6) $$\varepsilon_{app}=\frac{U_{app}}{4R\sin\left(\frac{\alpha }{2}\right)+2L\cos\left(\frac{\alpha }{2}\right)}$$

Though maximum principal strain usually cannot be achieved in the explicit form, its peak value in the interconnect of serpentine is related to the geometric parameters as well as the applied strain expressed as:

(7) $$\varepsilon_{\max-nonlinear}=\varpi F_{2}\left(L,\alpha ,\varepsilon_{app}\right)$$

where the membrane strain is ignored, *F*_2_ (*L*, *α*, *ε_app_*) is a function that is fixed numerically with an approximate model that depends on the finite deformation theory.

Fractal designs with stepwise iterations of the basic units have been used to achieve high stretchability in applications, such as epidermal sensors [88,318,322]. Fractal layouts with similar self-design also provide high stretchability with improved area coverage [88,323], as illustrated in Figure 6d. To realize the stretchability and deformation of fractal geometry, some theoretical models have been developed. Fan et al. [89] have demonstrated the deformations of different categories of fractal design with the FEM (finite element method) for pursuing the experimental evaluation. Figure 6b,c illustrate the comparison of optical images for the FEM and experimental results of second and third-order structures of Peano gold Nano membrane with the stretching at different levels, respectively. Figure 6e presents the fractal-inspired layouts to integrate hard–soft materials. Row (i) indicates the six different patterns of metal wires fully bonded to the elastomeric substrates. These patterns include the geometry of line, loop, and branch, omitting the sharp corners of arc sections to enhance the elastic mechanics with the assistance of mathematically explained fractal layouts. Row (ii) illustrates the FEM images for each (von Koch, Peano, Hilbert, Moore, Vicsek, and Greek) structure under the elastic tensile strain, and row (iii) indicates their corresponding MicroXCT images to exhibit the experimental elastic mechanics. Several analytical models have been developed to determine the elasticity in fractal interconnects. For example, Zhang et al. [323] introduced self-similar analytical models by establishing the recursive formulae at various fractal orders. These analytical models agreed well with the finite element analysis that demonstrated elastic stretchability [324].

### 5.3. Fabrication and Implementation

**Figure 7:**
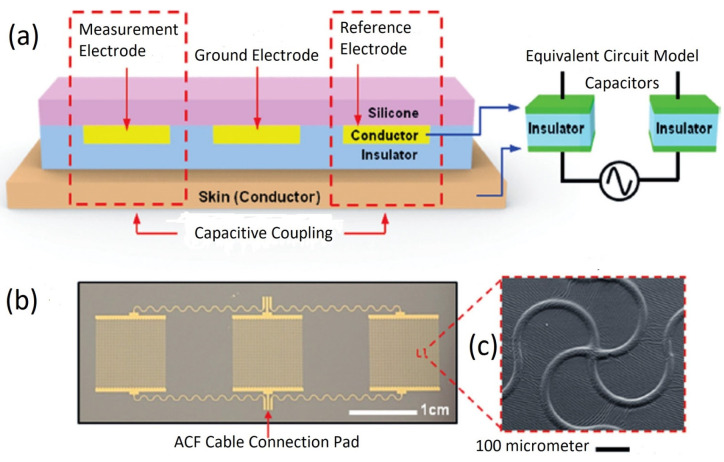
(**a**) Illustration of a capacitive sensor used to measure electro-potential signals and the equivalent circuit model. (**b**) Optical image of the capacitive sensor. (**c**) Scanning electron microscope (SEM) image of a unit cell of the filamentary serpentine mesh electrode structure. Reproduced with permission from ref. [18] Copyright 2013, Wiley-VCH.

Signal sensing with resistive electrodes from the human body brings electrical safety concerns to biological tissues and may cause allergic reactions and skin irritation, resulting in the feeling of discomfort in long-term usage. Such categories of electrodes face greater motion artifacts compared to capacitive electrodes. Electrode materials in capacitive electrodes are usually protected and encapsulated from the environment. Primarily, the signal quality at acquisition is measured by the coupling capacitance between the electrodes and tissues. High coupling capacitance is desired to achieve a better signal quality. The utilization of materials with a high dielectric constant or very small thickness offers a good solution. Jeong and co-workers [18] previously introduced a capacitive signal detection system for electromyograms (EMGs), electrocardiograms (ECGs), and electrooculograms (EOGs), where materials are protected from the surrounding environment with full encapsulation. Figure 7a illustrates the three fabricated capacitive electrodes as the reference, ground, and measurement electrodes that were coupled to human skin through an elastomeric soft insulating layer to detect the signals. The resulting signals were collected as measured voltages by a preamplifier with high input impedance. Figure 7b depicts the optical image of such a sensing system that can naturally obtain conformal contact with irregular, rough, and soft skin surfaces. Due to the elasticity and low modulus, the fabricated sensor avoided the constraints associated with natural skin motions. The constructed sensor consisted of three Au electrodes with 200 mm thick silicon, interconnecting wires, and anisotropic conductive film (ACF). Figure 7c shows the mesh layouts of filamentary serpentine (FS), where the metal for interconnections was encapsulated between the top and bottom layers of PI to position the metal in the neutral mechanical plane (NMP). This specific layout reduced the bending stresses of the active materials. Further, the contact pads could accommodate the bonding with ACF and consolidate the sensor to the measurement circuit for signal acquisition. The performance of the capacitive sensor usually depends on the geometrical layout of electrodes and the appropriate selection of the electrode materials. The second-order serpentine structure is preferred to enhance the stretchability and areal coverage. Ultrathin construction of materials with NMP configuration can improve skin contact and stretchability, resulting in fewer motion artifacts and better SNR.

A biocompatible and transparent insulating layer with large stretchability and high permittivity is strongly demanded in capacitive sensors to maintain good skin adaptability, strong skin lamination, and adhesion force for signal coupling. The thin insulating layer is usually chosen to achieve better conformal contact with the designed electrode. Additionally, it offers a practical encapsulation that normalizes the sterilization of the sensors to prevent electrode deterioration. Sterilization is a process that removes electrodes from the skin, which could be accomplished through a combination of heat, chemicals, irradiation, high pressure, and filtration methods such as steam under pressure, dry heat, ultraviolet radiation, gas vapor sterilants, chlorine dioxide gas, and so on skin facilitating the reuse of electrodes. In [18], the developed electrodes were initially attached to the right forearm for EMG measurement, and were retrieved with the help of a water-soluble tape (3 M, USA) as a supporting substrate and then sterilized with a 70% isopropyl alcohol swab (Dukal, USA) before being applied to the chest to collect ECG signals. The conformal skin contact succeeds only when the interfacial contact adhesion energy is larger than the total bending energy that comes from the electrode (membrane energy) and the elastic deformation energy of the skin [325]. The conformal contact enhances the accuracy of the signal measurement. The electrode adheres to the surface of the skin that is only driven by the van der Waals interactions; therefore, the traditional capacitive sensor can detect motion artifacts when movement occurs [326]. This is due to air gaps at the skin–electrode interface and the rigidity of electrodes. The issue could be resolved using soft, low modulus, thin, and stretchable/flexible electrodes to reduce the effects of deformation of the skin. Among the commercially available substrate materials, Solaris provides low elastic modulus, moderate relative permittivity, high elongation at breakage, and large adhesion force [18]. It is demonstrated in [18] that the activation timing of capacitive ECG electrodes (r ≈ 98%) is very close to that of Ag/AgCl (r ≈ 99%) for the direct electrodes. Additionally, such capacitive ECG electrodes also possess similar “PQRS” characteristics as these electrodes in the long-term recording. The same capacitive electrodes have been employed for EMG signal measurements during various hand motions and yielded almost similar amplitude as Ag/AgCl and direct electrodes (discrepancies have arisen for various positioning on the arm). This study highlights the potential use of such electrodes in research and clinic labs for the diagnosis of neuromuscular disorders [327], control orthotic and prosthetic devices [328], and muscle pain studies [329,330]. The obtained signal characteristics for EOG during the periodic changing of eye movements were comparable to conventional gel and direct electrodes, where the same capacitive electrode pair were placed near the right and left eyes. The resulted capacitive sensor provided the enhanced wearability level, sterilization and reusability, minimized artifacts, and lower leakage currents.

Dong et al. reported a stretchable bio-potential electrode in a self-similar serpentine structure for long-term, continuous, and stable recording of ECG signals via capacitive coupling from the human body onto the chest [21]. It has good conformal contact with the skin surface. Their designed electrodes were second-order self-similar structures with 40% stretchability and >30% deformability. The designed electrodes revealed that the multilayer structure (PDMS/PI/Au/PI) placed gold (Au) at the neutral plane to enhance the stretchability. The structure with the PI layer to the skin formed a capacitor. The result demonstrated that the fabricated bio-potential electrode exhibited good conformability to the skin surface via the Van der Waals force and good mechanical adaptability to the curved skin surface in long-term signal recording compared to the commercial Ag/AgCl electrode. The electrode performed well with the body motions without any delamination or constraints and provided a substantial amount of compliance when mounted on the chest. As such, the conformability reduced the motion artifacts that enhanced the stability, SNR, robustness, and susceptibility for signal recording even when an external load (walking, body swing, and running) was applied. This addresses the long-term wearability of electrodes. The measured leakage current was also less when compared to the Ag/AgCl electrode. A leakage current is an electric current in an unwanted conductive path under normal operating conditions. When two conductors are separated by a non-ideal dielectric, a leakage current will appear between the conductors. The leakage current is caused by a parallel combination of capacitance and dc resistance between the electrode materials and human skin. The leakage current appears when the dc resistance is insignificant compared to the ac impedance of various parallel capacitances. This current is a great challenging issue in biological applications because it creates a short circuit between devices/electrodes and the human body. In the capacitive design, an extra dielectric layer is placed between electrode materials and human skin, which enhances the safety of the patient, eliminating the leakage currents and hence short circuits between biology and electrodes. This layer produces an extra capacitor between electrodes and biology compared to the commercially available wet Ag/AgCl electrodes system [124]. So, the measured leakage current in capacitive design is less when compared to the Ag/AgCl electrodes [21]. Such property of electrical safety of electrodes not only saves biological cells of the skin, but to some extent, also protects organs, such as the heart and the brain. In general, a current of more than 0.5 mA is not allowed to flow into the human body from the wearable device [18] based on the guidelines of IEC (International Electrotechnical Commission) 60601-1; therefore, this study inspires the use of capacitive electrodes in research and clinical labs for monitoring heart diseases in patients with heart conditions. The lightweight and ultrathin nature of the electrode allows for comfortable use when laminated onto the body.

**Figure 8:**
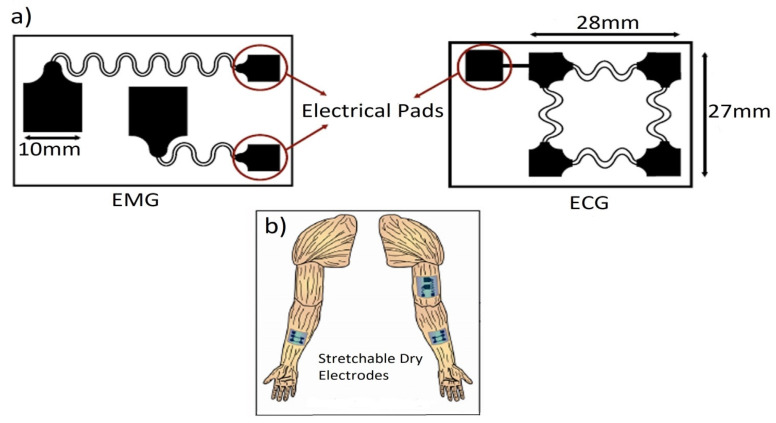
(**a**) Illustration of designed electrodes; EMG and ECG, where the black regions represent the skin pads attached to the externally electrical pads through the electrical interconnect of spring shape. (**b**) The positioning of electrodes on the human body. Reproduced with permission from ref. [22] Copyright 2019, Elsevier Publishing Group.

Shahandashti and co-workers [22] designed two stretchable electrodes to capture ECG and EMG signals from the human skin, as shown in Figure 8a. The designed electrodes were fabricated using a commercially available inexpensive flex substrate (Pyralux AP 9111) of copper-clad polyimide laminate (Cu (35 um)/PI (25 um)/Cu (35 um)) [331]. The designed EMG electrodes were adopted with the recommendations of SENIAM (surface electromyography for the non-invasive assessment of muscles) [332]. The fabrication process of designed electrodes was illustrated in [22]. A commercial Au plating method was used for electroplating an Au layer with a thickness of 6 µm on both sides of the substrate [333]. Then, using Edinburg [334] and Aqua Regia etchants [335], the Cu and Au layers were removed from one side of the entire structure. Using patterned Au and Cu as the hard mask, PI was dry etched. An adhesive tape was attached to the other side of the structure to protect the sample during the electroplating process. This side was covered with PDMS to enhance the mechanical performance due to its low stiffness [334]. The metal layer and PDMS usually improve the mechanical properties of the fabricated layers, such as cycling fatigue [331]. As a result, the electrodes exhibited a good mechanical deformation with more than 25% elongation. Moreover, Au and Cu layers were shaped in the form of springs to obtain good stretchability. A temporary glass substrate was employed to easily handle the sample. In this work, the Cu layer served as the key conducting material of the electrodes and the Au thin layer was placed on top of the Cu layer to enhance the biocompatibility with the skin. Figure 8b depicts the placement of the two electrodes in the arms and bicep for detecting ECG and EMG signals, respectively. A PI layer of 25 µm was employed for the relatively high-temperature soldering of external wires and to enhance the mechanical strength of the electrode due to its high tensile strength, excellent flexibility, very low creep, and good biocompatibility. The performance of these fabricated flexible capacitive electrodes was compared to the wet Ag/AgCl electrodes and provided greater comfort, accuracy, and less skin irritation.

**Figure 9:**
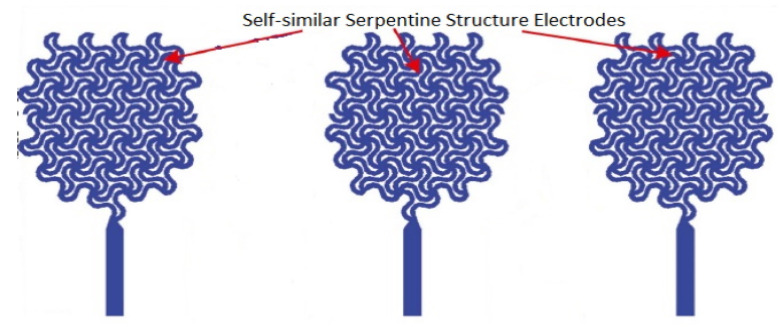
Illustration of self-similar serpentine structure electrodes for second order. Reproduced with permission from ref. [48]. Copyright 2021, Elsevier.

Dong et al. [115] fabricated stretchable sEMG capacitive electrodes with a self-similar serpentine configuration for long-term, continuous EMG signal monitoring. This structure provided large deformation (>30%) to quantify a high areal coverage, as validated by experiments and FEM simulation. The areal coverage, α is varied with the changing of dielectric (PI) layer thickness (*h_PI_*), area of the electrode, and capacitance in skin–electrode interface. The resulting electrodes were fixed on the surface of the skin conformally via van del Waals interactions, which reduced motion artifacts caused by body movements, enhanced wearability, and reusability, and expanded the electrode sterilization. The designed sEMG electrodes (ground, reference, measurement) to quantify the signals from the human body through a PI dielectric layer at the skin–electrode interfaces, where the line width of the electrodes was 50 µm and the node connection of the unit cells in the electrodes formed triangular lattices. The developed electrodes could also minimize the leakage current from the body by isolating the electrodes. The electrode network is comprised of horseshoe building blocks with hexagonal structure in the self-similar serpentine interconnect, illustrated in Figure 9, where the unit cells of the electrode network form the triangular lattices by connecting the nodes. The fabrication of designed electrodes was performed as the multilayered process on a silicon wafer by micro-electromechanical systems (MEMS) with Poly(methyl methacrylate) (PMMA) sacrificial layer. The PDMS on the glass substrate was used to pick up the electrode easily from the glass substrate after dissolving the sacrificial layer of PMMA in acetone. The complete structure (PI/Au/PI) (1.8, 0.3, and 1.2 µm approximately) is patterned as the stretchable form released from the wafer and transferred to the PDMS substrate. The fabricated electrodes were peeled off the laminate from the glass substrate with the aid of polyvinyl alcohol (PVA), which reduces the surface tension at the glass/electrode interface. The resulting electrode after PVA dissolution, adhered conformally to the skin surface to monitor and record the signals and provided large deformability and high areal coverage.

**Figure 10:**
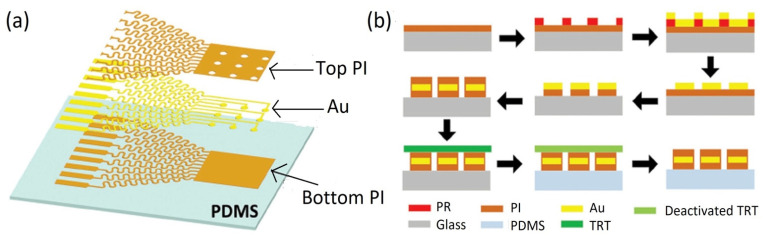
(**a**) Schematic view of the designed stretchable capacitive neural electrode array with a multilayer structure. (**b**) Illustration of fabrication steps for neural electrodes array on PDMS by thermal release transfer technique using TRT. Reproduced with permission from ref. [116]. Copyright 2017, Wiley Publishing Group.

We have focused our discussion on non-invasive capacitive electro-physiological sensors, while it is possible that sensors are created for implantable uses, such as electrocorticography (ECoG). For example, Yan and co-workers [116] developed soft stretchable capacitive electrode arrays with nine channels, which were mechanically tuned for brain tissues to enhance the development of brain disease diagnosis and brain–computer interface (BCI) systems. Their designed electrodes, illustrated in Figure 10a, had serpentine interconnect with a three-layered (PI (1 µm)/Au (80 nm)/PI (0.7 µm)) structure on the PDMS substrate to quantify the ECoG signal. In this study, the Au layer consisted of three modules: (i) output (nine parallel pads to connect to the ACF cable for ECoG recording), (ii) interconnectors (serpentine lines of width 100 μm), and (iii) input (nine small circles are connected to the output through the interconnectors). They employed a thermal release transfer printing technique to fabricate the electrode array with the aid of TRT (thermal release tape), which was utilized as the stamp to replace the traditional thick stamp (elastomer). The used TRT could pick up the fabricated electrode easily compared to the traditional transfer printing stamp due to its better adhesion ratio. This strategy provides an easy way to pick up micro devices from the substrate in the future fabrication of stretchable bioelectronics with more manufacturing convenience, less fabrication cost, and shorter time. Figure 10b shows the fabrication steps by the thermal release transfer printing technique. The developed PI/Au/PI structure remained in good condition during the entire heating process. The experimental result indicated the clear benefits of reducing the thickness of PDMS substrate to 50–720 µm to the extent of the conformal coverage and evaluating mechanical and electrical properties of as-developed electrode arrays indicating the potential utilization of such electrodes array or devices on brain tissue. This work is significant as it may enable physicians to quantify the ECoG signals accurately from the brain through the neural interfaces with good conformal contact to achieve an accurate diagnosis the brain diseases. Furthermore, this work also broadens the application scopes of flexible/stretchable capacitive electrodes for long-term signal monitoring or recording. It is also expected that the thermal release transfer printing technique will play a more important role in the manufacturing of other stretchable micro-devices, including wireless bioelectronics devices, skin sensors, and stretchable health monitoring devices.

**Figure 11:**
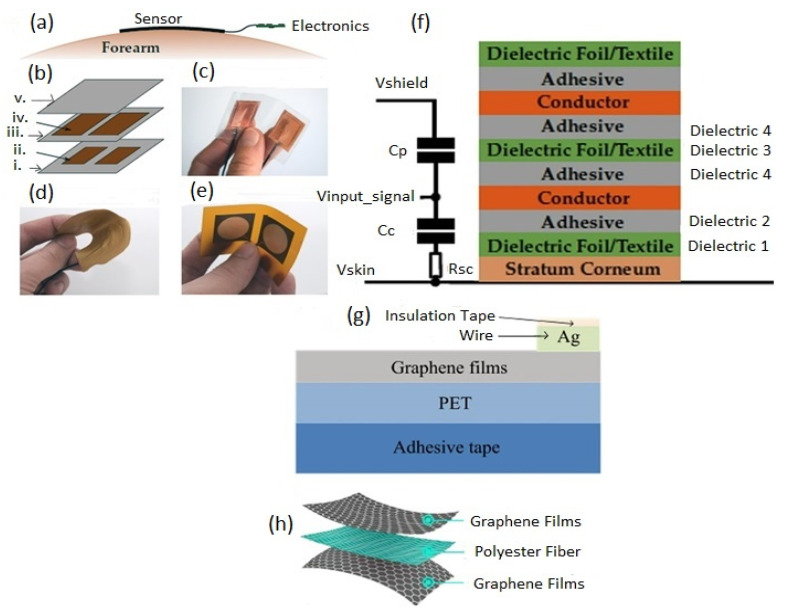
(**a**) Adaptation of the flexible sensor with the anatomy of the human forearm. (**b**) The multilayer structure of a sensor; ((i) dielectric 1, (ii) sensor areas, (iii) dielectric 3, (iv) shielding, and (v) dielectric. (**c**) Digital photographs of sensor 1 (copper sensor). (**d**) Sensors 2 and 3 (textile sensors). (**e**) Sensors 4–6 (flex sensors). (**f**) Stacking of sensor assemblies in detail (adhesive layers are acrylic and silicone, not covered in all assemblies of sensors). (**a**–**f**) Reproduced with permission from ref. [336], Copyright 2019, MDPI. (**g**) Schematic illustration of the graphene-based flexible capacitive electrode. (**h**) Schematic illustration of the graphene-based textile electrode. (**g**,**h**) Reproduced with permission from ref. [337], Copyright 2016, MDPI.

Our discussion of wearable sensors has been focused on flexible and stretchable designs; however, for some systems and applications, the focus is solely on flexibility. For example, Roland and colleagues [336] developed a capacitive flexible EMG sensor for stable and long-term signal detection for prosthesis control. The developed capacitive EMG sensor was found to be less sensitive to skin conditions (such as sweat) as well as body motion artifacts compared to state-of-the-art flexible sensors for prosthesis control. As seen in Figure 11a, the copper foil sensor was stacked on flexible materials, such as plastic foils, insulating and conducting textiles (textile sensors), and printed multilayer flex (flex sensors), so that it could properly adapt to the anatomy of the human forearm. Figure 11b displays the multilayered structure of such a sensor. This sensor quantified the signal in differential mode, such that one module comprised two symmetrical areas of the sensor. The air gap between electrode and skin was minimized to achieve better signal coupling. A shield was employed to protect the sensor from interference and enhance the common-mode rejection ratio (CMRR). For foil and textile sensors, wire shielding is often accomplished using UMCC (ultra-miniature coaxial cables) having an outside diameter of 1.37 mm. While flex sensors are multilayer flex printing (one layer is for shielding and the other layer for sensor area). There is no need for a separate wire because the sensors are connected to the electronic circuit in a direct line. As seen in Figure 11c, sensor 1 (copper sensor) had two adhesive layers with an individual thickness of 30 µm between a dielectric (silicone or acrylic adhesive) and a conductive layer, as shown in Figure 11f. Conductive layers are developed with the aid of self-adhesive copper tape. Sensor 2 (textile sensor) is comprised of conductive textile and dielectric layers narrowed for bonding, as shown in Figure 11d. Sensor 3 (textile sensor) is made of a narrowed multilayer textile.

For sensors 4–6 (flex sensors) (Figure 11e), the adhesive layer in flex prints was deleted even though its thickness was less than 10% of the total thickness of the flex prints. While sensors 4 and 5’s sensor regions were covered in dielectric and electrolytic gel, sensor 6’s sensor areas were coated in conductive material. In Figure 11f, R_sc_ is the stratum corneum impedance (assumed as 3 MW ohmic resistance), C_c_ is the coupling capacitance between electrode and skin, and C_P_ is the parasitic capacitance in differential mode due to the shielding of the sensor. Lou et al. [337] developed a graphene-based flexible capacitive ECG electrode for long-term monitoring by depositing graphene films on a flexible polyethylene terephthalate (PET) substrate with a thickness of 280 µm. The developed electrode exhibited high detection sensitivity, high flexibility, good biocompatibility and wearability, effective electrical performance, good comfortability, and high SNR at various motion states over time. Figure 11g presents the structure of the graphene-based capacitive ECG electrode. To fabricate this electrode, graphene films were first prepared on copper foils and then transferred to the PET substrate. The electrode was then connected with a silver wire using the conductive pulp of silver. The electrode and the conductive node were subsequently encapsulated with an insulating glue to protect the skin from direct contact with the electrode. Figure 11h depicts the structure of the designed graphene textile electrode, where graphene layers were deposited on the bottom and top surfaces of flexible polyester fiber. A plastic fastener was passed through the middle of the graphene textile and connected to the underlying surface using a metal snap for the external connection of the electrode. The developed electrodes could also be employed in other bio-potential sensing techniques, such as EEG and EMG.

**Figure 12:**
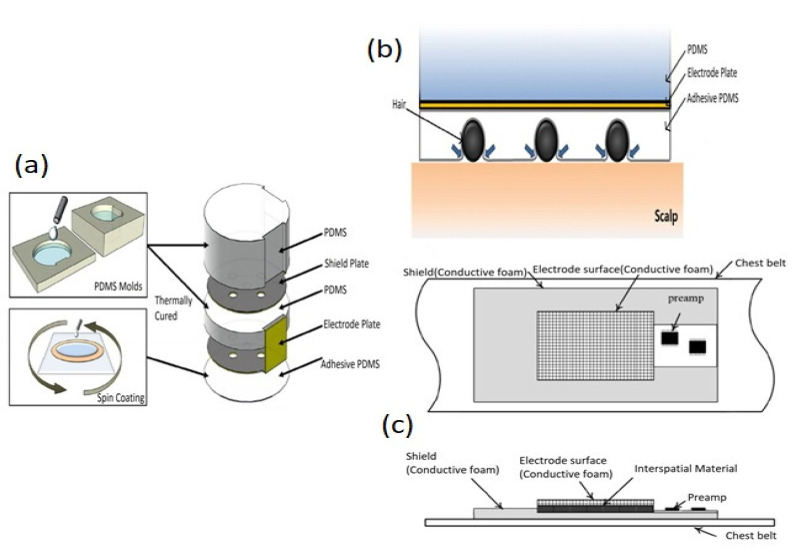
(**a**) Illustration of the assembly process of an electrode. (**b**) Cross-sectional view of a developed electrode attached to scalp. (**a**,**b**) Reproduced with permission from ref. [111]. Copyright 2013, IOP Publishing Ltd. (**c**) Configuration of flexible capacitive electrode (upper part: top view, lower part: side view). Reproduced with permission from ref. [339]. Copyright 2014, MDPI.

Ng et al. [338] have previously compared six different types of textiles, including cotton, nylon, linen, PVC textile, rayon, and polyester to determine the best insulating textile to be utilized between electrode and skin in capacitive biosensors. Each textile has unique properties, including relative permittivity, moisture absorption, and thickness, which can change the capacitance between electrode and skin. The performance of a sensor is influenced by several factors, such as signal noise levels, consistency, measurement accuracy, and resolution. The experimental results provided by Ng and co-workers revealed that the high electrode-skin capacitance of a TEX-C (textile-insulated capacitive) produced low noise floor and high signal quality. Thin textile material with higher relative permittivity is recommended. Linen and cotton exhibited good quality or consistent EMG signals and low noise floors of 2 mV. This work inspires the replacement of the insulating layer at the electrode–skin interface with an appropriate textile material to improve current wearable capacitive sensors. These textile materials are inexpensive, hypoallergenic, and commercially available. Lee and co-workers [111] reported a capacitive, adhesive, and small-sized flexible electrode for long-term cap-free EEG monitoring. The experimental results revealed the high signal quality of the detected alpha rhythm, N100 auditory, and high steady-state potential to diverse stimuli. Additionally, the electrode was biocompatible, comfortable, and convenient to wear, rendering it suitable for long-term monitoring or recording. Figure 12a illustrates the complete assembly process of electrodes comprised of PDMS layers, a shield plate, and an electrode plate. In this electrode, the lower and upper PDMS layers were used to handle the electrode structure; the shield plate was employed to block external noises. Moreover, the electrode plate functioned to collect the signals, and the adhesive PDMS layer was used to provide better adhesion for the electrode and achieve close contact on the scalp. Each layer of PDMS (including adhesive PDMS) was bonded to another layer with O_2_ plasma. Figure 12b depicts the cross-sectional representation of the developed electrode when attached to the scalp. The electrode was deformed and adjusted properly to the curvature of the scalp due to its softness and flexibility through hair. Because of its small size, the electrode was hidden in the hair, resulting in a high-quality signal. The developed electrode was semi-permanent and electrically safe, with no need for glue or gel attachment.

Lee et al. [340] have developed a CNT-based capacitive electrode using adhesive PDMS to record EEG signals from the hairy scalp for long-term recording, which is more suitable for BCI applications. The CNT-based capacitive electrode exhibited good flexibility and conductivity and provided good conformal contact to the skin or hairy scalp for long-term recording due to its self-adhesiveness. Their experimental result demonstrated that the developed electrode could provide higher SNR (3.71 ± 0.17 dB) compared to Ag/AgCl electrode with good motion tolerance. No leakage current was observed by using this CNT-based electrode due to its high-volume resistivity during the signal measurement. The developed electrode was directly connected to a commercially available acquisition system (MP150, BioPAC Systems, Inc., USA) with a band-pass filter of range 0.5 and 35 Hz for measuring EEG in place of a designed ultra-high input impedance preamplifier. It achieved a high-quality signal with an SNR of 3.71 ± 0.17 dB, whereas the fabricated electrode without CNTs and preamplifier exhibited poor SNR (2.79 ± 0.13 dB); therefore, the utilization of CNTs in a PDMS matrix could improve signal quality. The standard deviation of SNR with the electrode without CNTs for signal recording was 5 ± 0.77, approximately 1.4 times higher than that prepared using CNTs. The significant deviation without CNTs was caused by the increased prevalence of motion artifacts. The capacitive measurement was achieved with a gap (including air and hair) between the insulated metal layer and scalp; however, herein, the gap was filled up with the PDMS/conductive CNT, which induced high impedance between scalp and metal layer, thus leading to the good signal quality. The developed electrode is comprised of three components: PDMS/CNT, disk layer, and a PDMS ring. The disk layer consists of SU-8. The bottom surface of the disk was covered with bridges and cylindrical pillars to enhance the contact area of electrodes. The whole bottom and top surfaces of the disk are also roofed with a layer of Ti/Au and a Parylene C. Both conductive Ti/Au surfaces were connected through four holes. The layer of Parylene C prevented the direct contact of CNT/adhesive PDMS and conductive layers with the scalp or skin by forming a capacitive coupling. Lee et al. [339] have also presented a flexible capacitive electrode to acquire ECG signals via a wearable ECG device with minimized motion artifacts for long-term monitoring. Figure 12c shows the configuration of the fabricated capacitive electrode. The electrode is composed of gold-coated copper or only copper on the surface, a shield, a preamplifier circuit, and an interspatial material between the shield and electrode surface to maintain a fixed distance. The interspatial material had to be flexible while remaining noncompressible in the thickness direction. Urethane rubber was chosen as the interspatial material herein. The electrodes are employed with a chest belt to achieve the signal. The shield is placed between the chest belt and interspatial material, shown in the top view of Figure 12c. The experimental results demonstrated that the fabricated electrode was properly adapted and bent to the chest curvature even during walking or running up to a speed of 7 km/h. The conductive foam helped to minimize air gaps between the electrode and body surface and result a stable body contact of the electrode with fewer motion artifacts for long-term monitoring. It is possible to measure the signal with this capacitive coupling method over the clothes; therefore, patients can easily carry such sensors over their clothes and take them off. A bias resistor of 5 GΩ was utilized to further reduce the effects of motion artifacts, although its value varied with the contact pressure, cloth thickness, material, and external environment (such as atmospheric humidity). A PDMS film with micro-pillar arrays (mushroom-shaped) has also been developed as both the dielectric or insulating layer of a flexible capacitive dry electrode to detect bio-potential signals and the flexible adhesive patch on the electrode was attached to the body for long-term signal monitoring or recording [341]. Such integration could solve the existing shortcomings of medical adhesive patches and conductive gel electrodes by collecting and measuring electrophysiological signals from the body. The signal quality achieved using this flexible capacitive electrode is comparable to that acquired using the wet Ag/AgCl electrode with the adhesive medical patch. Compared to earlier efforts, where the investigations only focused on the dry (flexible/hard) adhesive medical patch or dry (flexible/rigid) capacitive electrodes, this integration offers a low-cost and straightforward technique.

**Figure 13:**
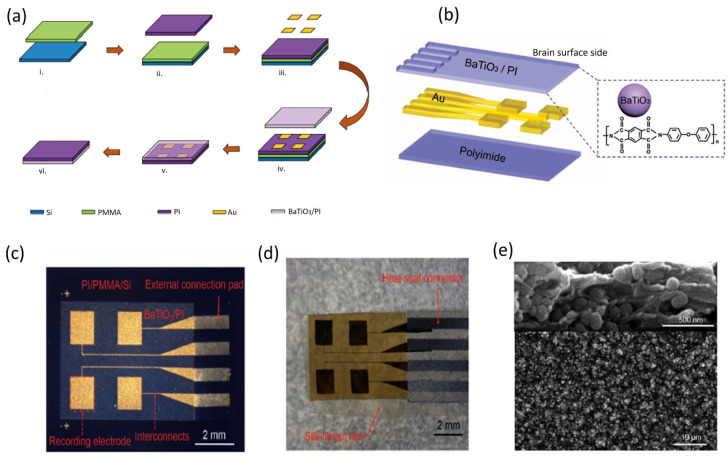
(**a**) Schematic illustration of the fabrication process of the BaTiO_3_/PI capacitive electrode array. (**b**) Illustration of the BaTiO_3_/PI capacitive electrode array showing the layered structures. (**c**) Microscopic image of the BaTiO_3_/PI capacitive electrode array before the dissolution of the sacrificial PMMA layer. (**d**) Microscopic image of the BaTiO_3_/PI capacitive electrode array after the transfer onto the silk fibroin film and linking to HSC. (**e**) SEM image of the surface of the as-prepared BaTiO_3_/PI composite film (bottom) and the cross-sectional SEM image (top). Reproduced with permission from ref. [342]. Copyright 2017, Wiley-VCH.

A new capacitive electrode array that employed a polymer composite, BaTiO_3_/PI (barium titanate/polyimide), as the insulating or dielectric layer in the capacitive electrode for ECoG signal recording has been reported [342]. Because of its high dielectric constant, the BaTiO_3_/PI composite was used as an insulating material. Figure 13a displays the fabrication process of the BaTiO_3_/PI capacitive electrode array, and the developed electrode array’s layered structures are depicted in Figure 13b. Figure 13c shows a microscopic image of the BaTiO3/PI capacitive electrode array before removing the PMMA sacrificial layer. Figure 13d shows the BaTiO_3_/Pi capacitive electrode after being transferred onto the silk fibroin film and jointed to HSC (heat seal connector). Generally, the increasing proportion of BaTiO_3_ in BaTiO_3_/PI, increased the dielectric constant of the composite due to the increased likelihood of agglomeration when the proportion of BaTiO_3_ became too high; therefore, 40 wt. % of BaTiO_3_ in BaTiO_3_/PI composite was selected as the optimum amount to ensure good dielectric properties and dispersion. To enhance the uniformity of BaTiO_3_ in the composite, 3-aminopropyltriethoxysilane (APTS) coupling agent was added to BaTiO_3_. The SEM image of the composite showed the uniform distribution of BaTiO_3_ throughout the PI matrix after imidization, and no aggregation of BaTiO_3_ was observed in the composite (Figure 13e), thus revealing the excellent compatibility between PI and APTS-modified BaTiO_3_. Further, a comparison of the X-ray diffraction (XRD) patterns of pure BaTiO_3_ particles and BaTiO_3_/PI composite showed no noticeable difference. This finding indicated the stable crystal structure of BaTiO_3_ during the composite preparation. The energy-dispersive X-ray spectroscopy (EPS) mapping of the BaTiO_3_/PI composite confirmed the presence of Ti, Ba, and O elements, thus indicating the successful incorporation of BaTiO_3_ particles into the PI. The signal quality generated by such an electrode array is comparable to the resistive electrode array and conventional screw electrodes, even to a pure PI capacitive electrode array. It is also observed that the signal recording quality for pure PI electrode array is worse than BaTiO_3_/PI composite capacitive electrode array and conventional screw electrodes (where the anchoring screws are used to secure the electrodes to the skull). When a current of 5 mA is passed through the electrode, the developed BaTiO_3_/PI composite capacitive electrode array produces lower leakage currents of several nA compared to the resistive electrode array; therefore, this composite electrode array could provide improved electrical safety for biological tissues than the resistive electrode array. Moreover, it possessed good conformability to biological tissues owing to its ultrathin thickness of 4.2 µm and good flexibility.

From the studies presented in this section, it can be concluded that: first, four categories of electrode-skin models with equivalent circuits are capable of addressing the physics or mechanism of signals transduction among the various types of electrodes and skin. Second, the adaptation of electrodes to the curvilinear human skin of the human body and the quality of the detected signal depends on the size and thickness of the electrodes and insulating layers. The coupling capacitance between electrode and skin significantly varies with the changing of size and thickness of electrodes and insulating layers. The conformal contact of sensors to the skin highly depends on the electrode design. Specifically, the electrode design influences the coupling capacitor value that has a significant impact on the quality of the signal to be measured. Consequently, the proper design of the electrode is highly critical. One can easily design effective capacitive electrodes by utilizing the strategies discussed in the design section. Third, the same fabricated electrode could be employed to collect all categories of electrophysiological signals such as ECG, EEG, EMG, and EOG, depending on the placement of electrodes in the various part of the body. It is also possible to achieve higher quality signals during body motions by fabricating electrodes with good adaptability to the skin, which is difficult to achieve with commercially available gel electrodes.

## 6. Body-to-Electrode Signal Transduction and Measurements

**Figure 14:**
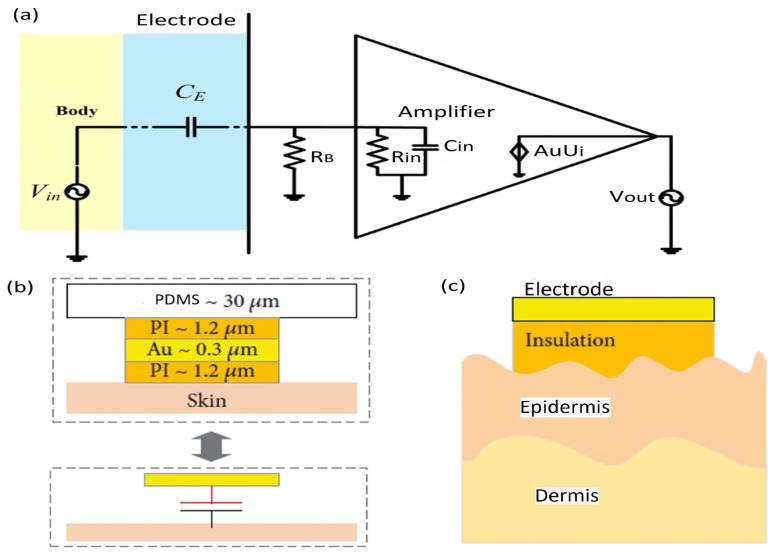
(**a**) Equivalent electrical model for a capacitive sensing electrode with a voltage follower. Reproduced with permission from ref. [341]. Copyright 2014, Elsevier. (**b**) Electrode with multilayer structure through capacitive coupling. (**c**) Wrinkles of the stretchable electrode with the skin surface. (**b**,**c**) Reproduced with permission from ref. [346], Copyright 2018, Wiley-VCH.

Biopotential signals are created by using the different ion concentrations (Na^+^, Cl^−^, Ca^2+^, and K^+^) between the interior and exterior of muscle cells [343]. The produced signal is transferred to the skin surface via ions and quantified by placing the electrodes on the skin. The electrode–skin impedance plays a major role in determining the quality of the measured signal. Low electrode–skin impedance is desired to achieve a high SNR. High electrode–skin impedance enhances the susceptibility of interference and motion artifacts due to body movements and decreases the signal amplitude, thereby leading to a low SNR. This implies that good electrode–skin contact is important to ensure good signal quality. Flexible and stretchable capacitive electrodes could ensure good electrode–skin contact. In addition, they also reduce the possibility of leakage current (electrical) from electronics to the body and body to electronics (fluidic). Leakage current is harmful to human tissues and patients with low immunity though it usually occurs at very low levels. It is highly essential to control the leakage current to prevent the soft tissues from being damaged during signal monitoring and recording. Capacitive electrodes can provide electrical safety, especially for the soft tissue of the brain and heart. This is due to the insulating layers in these electrodes, which provide effective encapsulation and facilitate cleaning for reuse and sterilization. Further, achieving low electrode–skin impedance in capacitive electrodes is challenging. The electrode–skin impedance in capacitive electrodes is larger compared to that in conventional Ag/AgCl electrodes due to the existence of insulating layers [344]; therefore, an impedance preamplifier or a voltage follower (buffer circuit) has to be utilized to reduce the effect of high impedance on the signal transmission or acquisition from the capacitive electrode. Researchers have tried to achieve low electrode–skin impedance by designing an external measurement system with the assistance of a basic electrical model for the capacitive electrode, as shown in Figure 14a. Various categories of electrical models have been proposed to understand the electrode–skin impedance behavior at the interface [345]. Figure 14a depicts a model of a capacitive sensing system with a voltage follower for an electrode to measure the signals from the body externally. Herein, the reduced leakage current was lower than 0.2 mA when a current of 10 mA current was passed through the electrodes. In this study, a bias resistor *R_B_* forms the charge–discharge path between the input of the operational amplifier (op amp) and the ground for the coupling capacitor, *C_E_*, between the insulated electrode and body skin. *R_B_* and *C_E_* form a first-order high pass filter with the parallel combination. *C_E_* measures the SNR of the collected signal through the effect of the gain of the amplifier. The transfer function or gain of the amplifier in the voltage follower design can be expressed by the equation:

(8) $$G(s)=\frac{V_{out}(s)}{V_{in}(s)}=\frac{sC_{E}R_{B}R_{in}}{R_{B}+R_{in}+s(C_{E}+C_{in})R_{in}R_{B}}$$

where *C_in_* and *R_in_* are the input capacitance and resistance of op-amp, respectively. The value of the bias resistor should be chosen carefully to make the cut-off frequency (f_c_ = 1/2π*R_B_C_E_*) to be less than 1 Hz to prevent the attenuation of the physiological signal [18,111,341]. If *C_E_* becomes too large compared to *C_in_*, then the signal is to be coupled as capacitive with near unity gain. A CMOS op-amp having ultra-high input impedance could be employed to drive the differential gain stage, which is configured as the voltage buffer with unity gain. The static charge at the skin–electrode interface was deposited effectively and could be reduced with the assistance of *R_B_*. When two surfaces are in relative motion, a static charge is formed. A bias resistor of 10 GΩ was employed to reduce the static charge, and a high-pass filter (passive RC) with a cut-off frequency of 0.7 Hz was utilized to avoid low-frequency body motion artifacts [341]. As a result, a stable high signal gain within the frequency range of 20–1000 Hz was achieved to enhance the signal quality of a capacitive electrode system with low input capacitance and high input impedance. The impedance of electrode–skin contact at the interface decreases with the increase in frequency. The input capacitance is expressed at the electrode–skin interface with a dielectric layer of PI as below:

(9) $$C_{S}=\varepsilon \alpha \frac{S_{S}}{h_{PI}}=\varepsilon \frac{S_{effective}}{h_{PI}}$$

where *S_s_* is the area of the electrode, *α* is the coverage of the electrode, *h_PI_* is the thickness of the dielectric layer of PI, and *ε* is the dielectric constant. The equivalent circuit model’s capacitances and resistances were used to define the interface properties. The dielectric layer and stratum corneum (SC) of the skin are equivalent to the RC parallel circuit. Figure 14b depicts the electrode with the multilayered structure of PDMS (~30 µm)/PI (~1.2 µm)/Au (~0.3 µm) and PI (~1.2 µm) along with the capacitive coupling. The electrodes with PDMS/PI/Au/PI structure ensure the Au layer in the neutral plane of the electrodes to enhance the flexibility of electrodes by reducing the strain of the Au layer. Figure 14c shows the stretchable electrodes illustrating the wrinkles on the surface of the skin.

The acquired signals by the developed electrodes are usually amplified, filtered (noise limiting), and digitized for converting analog to digital with the commercial instruments or an own developed system. Then the resulted signals are processed and analyzed with software to enhance the signal quality. For example, Shahandashti and co-workers [22] developed a signal measurement system for the same purpose that consisted of a band pass of 50–2500 Hz filter, an operational amplifier, an instrumentation amplifier, and a single lead heart monitor of single lead (AD8232 from Analog Devices^TM^). The system provided a gain of 100 dB in the frequency range of 0.5–100 Hz. The contact impedance at the interface of the electrode and skin was measured in a frequency range of 1 to 10,000 Hz with the fabricated flexible/stretchable capacitive electrode. Compared to the Ag/AgCl electrode, the contact impedance of capacitive electrodes is reduced gradually with time but not immediately similar to the Ag/AgCl electrode. This indicates the suitability of capacitive electrodes for long-term signal monitoring. Signal levels for EEG measurement and recording are more challenging compared to ECGs (0.05–3 mV) and EMGs (0.001–100 mV) due to their very small amplitude (10–100 µV). Such a low signal is easily affected by noises. The impedance changes with the applied pressure to the skin. Moreover, hairs between the scalp and electrode provide an obstacle to measuring the signals accurately due to the absence of good contact at the interface. The results show poor signal quality with a low dielectric constant. Further, EMG signal monitoring is more challenging compared to ECG due to its relatively wide bandwidth of frequency of 10 Hz–5 kHz, low signal amplitude, and greater dependence on the location of the electrodes. Moreover, the fabricated electrodes provide relatively poor sensitivity to the body movements resulting in less motion artifacts and better SNR compared to Ag/AgCl electrodes. The distortion is mainly imposed on the variation of electrode–skin impedance during body movements. An appropriate location for the electrode is necessary to improve the signal quality. An ECG measurement with a capacitive system demonstrated less sensitivity for the upper body swing compared to direct and wet gel electrodes [18]. Furthermore, the same signal amplitude and activation timing for EMG were observed from the forearm flexor carpi radialis to the motions of fingers and hands, but inconsistency arose when the signal was measured from different positions across the arms. The power spectra density (PSD) of the EMG signal measured from the forearm was concentrated in the region below 250 Hz [45]. Jeong et al. [18] also introduced an external signal measurement system for a capacitive sensing system with a voltage follower, filter, amplifier circuit, and high input impedance. In this study, SNR could be improved to provide the negative feedback of common-mode noise (residual) to the ground on the skin with the driven ground. The filter and amplifier units provided a tunable gain of 60–80 dB to measure the signals, whereas a 60 Hz notch filter could effectively reduce the interference of the power line. The experimental results demonstrated that gains of 60 dB, 80 dB, and 80 dB were achieved in the frequency range of 0.5–100 Hz, 10–500 Hz, and 0.5–20 Hz for ECG, EMG, and EOG, respectively, and further improvements could be made by increasing the coupling capacitance (*C_E_*), which is greatly influenced by the electrode size. A 5 µm insulating layer of PI provides a more stable gain over the interest range of frequency with the comparably high *C_E_* (~120 pF). Only a small leakage of 10 nA was observed when a current of 5 mA was passed through the structure, thus maintaining the IEC 60601-1 standard. The obtained signal quality was comparable to the direct contact system and wet Ag/AgCl electrode for the same electrode design. The performance of a capacitive sensing system could be directly compared to the direct contact of the electrode system and wet Ag/AgCl electrode using the Pearson correlation coefficient (r) [291]. Several used instruments and software features and purposes in the literature are described in Table 3.

**Figure 15:**
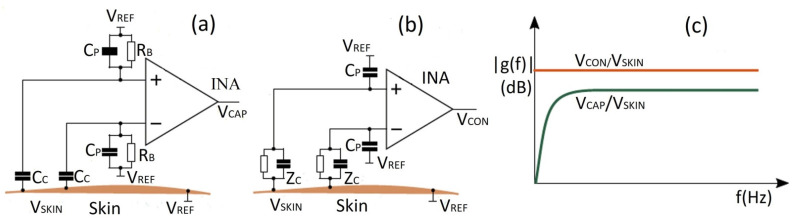
Comparison of conductive and capacitive signal measurement set-ups. (**a**) Capacitive Measurement. (**b**) Conductive measurement (ZC indicates the coupling impedance). (**c**) Transfer functions for both measurement set-ups. Reproduced with permission from ref. [336], Copyright 2019, MDPI.

Roland et al. [336] compared the conductive and capacitive measurement set-ups to interpret the current challenges in EMG measurements, as depicted in Figure 15. The amplifier stage in the input was accomplished with the CMRR of the instrumentation amplifier (INA) to reduce interferences. The direct current (DC) operating point is associated with the bias resistor, *R_B_*, for the capacitive set-up. For the conductive set-up, it is realized with the reference electrode. Within the amplifier operating range, a stable operation point is needed for signal acquisition. The coupling capacitance, *C_c_*, builds a voltage divider with a bias resistor, *R_B_*, parallel to the impedance of parasitic capacitance, *C_P_*. This provides a smaller amplitude signal of the voltage divider at the insulated electrode, whereas no bias resistor is needed in the conductive measurement. The path of bias current is introduced through conductive connection to the skin; therefore, the amplifier input impedance can be exploited. The DC potential of the signal is represented by a reference electrode, which establishes a conductive connection to the skin. In conductive measurement, higher amplitude and SNR of the signal are usually achieved compared to the capacitive measurement; however, owing to the high sensitivity of conductive sensors to skin conditions, such as sweat, these are more sensitive to body motions and patients feel discomfort due to skin irritation and allergic reactions. Hence, conductive measurement is not as suitable as a capacitive measurement for long-term signal monitoring or recording applications. Another shortcoming of conductive measurements is the strong possibility of leakage current due to the direct contact of electrodes to the body compared to the capacitive system. Due to the higher signal stability in capacitive sensing measurement, amplifier adaptation is not so essential in practical applications. With increasing parasitic capacitance, the CMRR increases, and the signal amplitude attenuates gradually. The CMRR can be adjusted automatically by setting an appropriate capacitance ratio. An appropriate capacitance ratio can be achieved by selecting suitable insulating materials for a sensor. As such, a capacitively coupled signal is usually collected and measured using a high-precision electronics circuit. The insulating or capacitive effect between skin and electrode is made insignificant with the ultrahigh input impedance preamplifier.

The received signals from the human body with the developed electrodes need to be analyzed to diagnose the findings from those signals. There are two ways to analyze the signals. In the first approach, the physicians could diagnose the signals manually to find whether it is bearing any disease or not. The second approach involves computer-aided automatic diagnosis. The first one is time-consuming and inefficient to some extent due to the natural limitations of human beings as well as the expertise and experience level of physicians. So, automatic diagnosis has been of great interest to physicians to handle a large number of patients within a short time and efficient diagnosis. This approach reduces the extreme workload on physicians and supports individuals in medical and clinics. A traditional automatic system consists of mainly three parts: (i) preprocessing, (ii) feature extraction, and (iii) classification. For instance, an automatic cardiac arrhythmia recognition system from ECG signals was proposed by Jha et al. [347], where feature extraction was performed with the tunable Q-wavelet transform mechanism and classification was accomplished with a support vector machine (SVM) classifier. There are various feature extraction algorithms available in the literature, such as LBP—local binary patterns, WT—wavelet transform, HOS—higher-order statistics, and EMD—empirical mode decomposition. Various classification techniques, such as artificial neural network (ANN), genetic algorithm (GA), random forest (RF), naive Bayes (NB), decision tree (DT), logistic regression (LR), k-nearest neighbor (KNN), Q-WT, and SVM, could be used to perform the classification function. The raw ECG signals are usually involved with various categories of noises such as baseline drift, power-line interference, and motion artifact noises. These noises influence the actual diagnosis of signals. Hence, before feature extraction and classification, these noises should be removed or reduced for accurate diagnosis in the preprocessing step (part-i). Various filtering mechanisms such as Kalman, Savitzky–Golay (SG), Butterworth, and discrete wavelet transform (DWT) have been used in the literature, considering the raw signal characteristics and quality and computational complexity as well. In [347], an SG filtering approach with the polynomial order of 5 and a window dimension of 21 was employed to remove the high-frequency noises. On the contrary, a Butterworth filter with an order of 3 and a cut-off frequency of 1 Hz was used to suppress the low-frequency noises. Similar processing will also be applicable for other electrophysiological signals such as EEG, EOG, ECoG, and EMG with consideration for the characteristics and variation of different bio-potential signals and techniques in different steps (preprocessing, feature extraction, and classification). Despite the fact that automatic systems with feature engineering techniques are widely used due to their low computation complexity, they still face several challenges such as dealing with class imbalanced data (all real-life data are class imbalanced), over-fitting of models, dealing with big data, and extracting features from complex wave shapes. Furthermore, the amount of data in medical and clinics is increasing day by day, as well as long-term monitoring of patients with wearable sensors, which features a tremendous emergence of wearable electronics, is also a significant issue for a large amount of data due to the real-time signals monitoring continuously. Deep learning approaches could be a good candidate to resolve the aforementioned challenges of traditional automatic systems (features engineering techniques) where no feature extraction step is available. The designed deep model automatically extracts the required features from the raw signals and then classifies them. The major shortage of deep models is that a sufficient amount of training data is required to optimize the model. Importantly, the amount of data is enlarged day by day in medical and clinics and wearable electronics fields. The deep learning models usually provide good interpretability and high classification accuracy in the literature compared to traditional automatic classification methods [123]. With the recent tremendous development of computer technology and networking, high configuration GPU (graphical processing unit), personal computer (PC), and cloud systems play a great role in the computational complexity reduction in deep learning models. Among the different deep learning networks, such as deep neural networks (DNNs), deep belief networks (DBNs), convolutional neural networks (CNNs), long short-term memory (LSTM), recurrent neural networks (RNNs), gated recurrent units (GRUs), and generative adversarial networks (GANs), CNNs are widely used to diagnosis the raw signals automatically. In CNNs, one-dimensional CNN (1D CNN), two-dimensional CNN (2D CNN), and three-dimensional CNN (3D CNN) consider the time-series raw or preprocessed signals, equivalent images of raw or preprocessed signals, and videos of raw or preprocessed signals, respectively, as its input. The 2D CNN provides better performance compared to 1D CNN [122,123]. Dong et al. [48] designed a soft multi-functional electronic skin (SMFES) to collect several vital signs (sweat and temperature) and EOGs from the human body for the detection of eye movement for wearable applications. Their designed sensors provide good stretchability (deformation > 30%), which is adaptable to the human skin. The collected EOGs signals and vital signs are first filtered with a band pass filter (0–20 Hz frequency) to remove the high-frequency noises, and then the continuous biological data from the sensors is divided into EOGs, temperature, and sweat/hydration signals using sliding window method based on the duration of eye movement during the repeated eye movements. An adaptive neuro-fuzzy inference system (ANFIS) classifies the biological data into three classes EOGs (based on the signal features and other biomarkers, temperature, and sweat/hydration signals with good accuracy (90%). The principal component analysis (PCA) technique was adopted to reduce the dimensionality of the feature space of collected signals. Similar way, the various categories of diseases such as cardiac arrhythmia (from ECG signals), sleep apnea (from ECG/EEG), epilepsy (from EEG), Alzheimer’s (from EEG), neuromuscular diseases (from EMG), pattern dystrophies (from EOG), musculoskeletal injuries, heart/brain diseases, and human activity recognition are also possible to diagnosis from a customized sensor [348,349,350,351]. 

From this review, several important points can be summarized: first, the measured signal quality is not only dependent on the size and thickness of the materials or properties of the electrodes and insulating layers but also on the signal measurement system. One can obtain the various quality signals for the same fabricated electrodes with custom-made measurement systems by considering the basic equivalent electrical model or with commercially available instruments and components. Second, commercially available instruments can be utilized to measure electrophysiological signals with the fabricated capacitive electrodes as well as some associated software to process the archived signals for achieving good signal quality. These signals could also be recorded and stored for further analysis to diagnose diseases. Third, the underlying mechanism behind impedance variation at the electrode–skin during signal transduction and measurement.

## 7. Conclusions and Future Outlook

Wearable sensors have received considerable interest in health monitoring to improve the diagnosis as well as the quality of life. These sensors can be used to obtain various information from the human body that can be utilized to diagnose diseases. The major challenge in commercialization lies in improving the conformability and flexibility of these sensors. In this review, we have focused on the recent development of wearable capacitive flexible and stretchable electrophysiological sensors. These sensors are developed by using different stretchable structures and soft, thin materials, which could provide improved and precise integration with the human body. As a result, the robustness, susceptibility, mechanical adaptability, and stability of soft curvilinear body surfaces are enhanced. Additionally, the artifacts which appear due to the body motion are also alleviated. To develop high-performance wearable sensors, proper design and selection of the electrode configuration, electrode materials, and fabrication strategies play important roles. A detailed discussion on these issues has been provided in this review. It is also possible to monitor and record the signals continuously for long-term health monitoring applications. The signal can be collected on a PC or server directly or wirelessly for further analysis, while signal processing, features extraction, and machine learning techniques play an important role. As a result, such sensors can contribute greatly to telemedicine and HMI (human–machine interface) as well as provide effective tools to diagnose various diseases. In these studies, we have discussed several developed capacitive sensors to monitor or record ECG, EOG, EEG, and EMG signals. Diversified wearable devices can be prepared by connecting the capacitive recording system to smart mobile phones, which can be marketed as healthcare products for consumers. Such wearable sensing devices will generate social and economic impacts. Moreover, several challenges have to be addressed, including (i) designing electrodes with good geometries and dimensions, (ii) ensuring a compatible selection of electrode and insulating materials, and optimizing the thickness of the insulating layer, (iii) ensuring stable attachment/positioning of electrodes to the skin surface during body movements, (iv) high impedance at the electrode–skin interface, (v) installation and packaging, and (vi) minimizing instrumental noises and electromagnetic interference. These challenges can be solved by: (i) Fabricating electrodes with good design and consolidated materials with layer thickness that can provide lightweight and adequate contact with the skin surface, (ii) having a high-precision circuit arrangement with ultra-input impedance (1 MΩ to several hundred MΩ) to address the challenge (iv); (iii) develop a miniaturized system to address the challenge (v), and (iv) an effective and efficient signal processing model to solve the challenge (vi). All challenges mainly introduce motion artifacts during body movements for long-term signal recording and result in poor SNR that can lead to an incorrect diagnosis; therefore, the use of effective/efficient machine learning models (features engineering or deep learning such as convolutional neural network (CNN), RNN (recurrent neural network), LSTM (long short-term memory)) may provide adequate solutions to all the above challenges. To achieve commercialization, step (iii) is a key factor.

Finally, we can conclude that despite several challenges in the practical implementation of wearable flexible/stretchable capacitive sensors for long-term electrophysiological signal monitoring or recording, they have great prospects in the healthcare field to improve our social life. A powerful compact system with such sensors can offer a good platform for long-term, stable, and continuous monitoring of health conditions. The review will provide useful guidance for future design and construction of wearable capacitive sensors for monitoring electrophysiological conditions in the human body.

## Figures and Tables

**Table 1:** Several widely used substrate materials in wearable electronics including their pros, cons, and Young’s modulus.

Substrate Materials	Pros	Cons	Young’s Modulus
PDMS [96,175]	Commercially available, cheap, biocompatible, transparent, non-flammable, low autofluorescence, chemically inert, and easy processing	Difficult to integrate electrodes on the skin, absorb small hydrophobic molecules, adsorption of proteins on its surface	0.5–3 MPa
PI [64,182,183]	Good wear and low creep resistance, low flammability, high thermal stability, high tensile strength, good flexibility, and infusible	Expensive, low impact strength, poor resistance to hydrolysis and alkalies, and attacked by concentrated acids	2.3 GPa
Ecoflex silicone [149,182,184,185]	Safe for skin, highly stretchable with low modulus, excellent printability, good transparency, and good heat and creep resistance	Poor tear strength, comparably high cost, ultimate tensile and tear are declined with thinner, and poor transparency	50–100 kPa
PMMA [186]	Excellent optical clarity, good UV and abrasion resistance, low temperature, good track and arc resistance, low fatigue, low smoke emission, low water absorption	Poor solvent and fatigue resistance, notch sensitive, limited chemical resistance, poor abrasion and wear resistance, cracked under load, prone to attack by organic solvents	2000 MPa
Polyamide (PA) [187]	High abrasion resistance, good thermal resistance, good fatigue resistance, high machinability, noise dampening ability	Water absorption, chemical resistance, high shrinkage, and lacks of dimensional stability	4750 MPa
Liquid Crystal Polymer (LCP) [188]	High heat resistance, flame retardant, good dimensional stability, moldability, low viscosity, adhesion, weldable, wide processing window, excellent organic solvent, and heat aging resistance	Weak weld lines, chemical resistance, high anisotropic properties, high Z-axis thermal expansion coefficient, less cost-effectiveness, and knit line strength	10.6 GPa
Thermoplastic polyurethane (TPU) [189]	Excellent abrasion resistance, good impact strength, rubber-like elasticity, toughness but good flexibility, good resistance with abrasion, oil, and grease	Short shelf life, less cost-effective, drying is needed before processing, easily degrades with sunlight or UV exposure, easy fracturing feature	3.6–88.8 MPa
Polyethylene terephthalate (PET) [190]	Inexpensive and available, high resistant to moisture, high strength to weight ratio, high chemical resistance to water and organic materials, highly shatterproof and transparent, easily recycled	Low heat resistance, resins and susceptible to oxides, lower impact strength, lower moldability, more sensitive to high temperatures (>60 °C), highly affected by tough bases, boiling water, and alkalis	2.5 GPa

Human skin-Epidermis 140–600 kPa, dermis 2–80 kPa [72].

**Table 2:** Several widely used flexible electrode materials including their advantages, disadvantages, applications, electrical property, and Young’s modulus.

Electrode Materials	Advantages	Disadvantages	Applications	Conductivity/Thermal Conductivity	Young’s Modulus
PEDOT [234]	Optically transparent, high stability, moderate band gap, and low redox potential	Poor solubility, acidity, anisotropic charge injection, hygroscopicity	Biomedicine (drug delivery, tissue engineering), wearable electronics (biosensors), industry (optoelectronic/thermoelectric devices, fuel cells)	1200 S/cm	2.6 ± 1.4 GPa
PANI [235]	Controllable and wide range of conductivity, transparent and colored electrically conductive products, environmental stability, reversible doping, and pH change properties, simple synthesis	Low processing capacity, inflexibility, lack of biodegradability, poor solubility	Renewable energy storage devices (Li–ion batteries, supercapacitors, Li–sulfur batteries), medicine (delivery systems, neural biotic abiotic/prosthesis interfaces, scaffolds), electrochromic glasses, electroluminescence	5 S/cm	2–4 GPa
PPy [236]	Biocompatibility, easy synthesis, the inspiration of proliferation and cell attachment, good electrical conductivity, environmental friendliness	Non-thermoplastic, brittle, rigid, non-degradable, and insoluble in some common solvents (for example: acetone, methanol, ethanol)	Optical, medical, electronics, electrochemical and biological applications (as the sensors), catalyst support of fuel cells, micro-actuators	40–200 S/cm	430–800 MPa
Polythiophene (PT) [237]	Low cost, good electrical, mechanical, and optical properties, high thermal and environmental constancy, smaller band gap energy (2.0 eV) compared to PANI and PPy	Poor solubility with ordinary solvents, hard to synthesize, poor chemical stability and processibility	Biosensors, solar cells, thermoelectric applications, OLEDS, FETS, batteries, memories, electroluminescent devices	10–100 S/cm	3 GPa
Graphene [238,239]	Mechanically strength, more energy storing for a long time and fast charging capability, lightweight, good thermal and electrical behavior, flexibility, chemically inert	An expensive, complex process that cannot be switched off easily, susceptibility of catalyst to oxide environments, toxic chemicals are required to grow it	Aerospace, mobile devices, building materials, heat sinks, microelectronics, batteries, fuel cells, supercapacitors, flexible solar panels, flexible displays, drug delivery, DNA sequencing	~4000 W/mK	1 TPa
Diamond [240]	Low affinity and friction coefficient with non-ferrous metals, high thermal conductivity, good quality machined surface, good anti-adhesion, excellent cutting performance, tool durability	Low thermal stability, poor toughness, chemical reaction contacting with iron group of elements, grinding of diamond tools is costly and difficult, carbonization at 700~800 °C	Industry, medicine, engraving, audio equipment, beauty products, heat sinks, medical devices, super lasers, surgical tools, windows, jewelry	1000 W/mK	≈103 GPa
Carbon nanofiber (CNF) [241,242]	Low density, good thermal stability, high modulus, large aspect ratio, high strength, high conductivity, compact structure ability	Lack of solubility with aqueous media, hydrophobicity, large surface area, insolubility, non-uniform morphological structure	Filtration, tissue engineering, nanocomposites, water treatment, packaging, sensing, energy devices, drug delivery, photocatalytic	2000 W/mK	6–207 GPa
Glassy carbon [243,244]	Reproducible features, high-temperature resistance, extreme resistance with chemical attack, versatility in miniaturized devices, excellent electrical, chemical, thermal, mechanical properties	Concoidal fracture surface, brittle, dimensional shrinkage, impermeability in liquids and gases, high costs in large-scale structure production	Antistatic packaging, Electrode material in electrochemistry, tissue engineering, electrochemical sensors, biomedical, energy storage sectors, pharmaceutical, encapsulation for nuclear waste	4.6–6.3 W/mK	20 GPa

**Table 3:** List of used instruments and software based on the literature reported.

Measuring Instrument	Features	Purposes	Used Software	Reference
Instrumentation amplifier: AD620 (Analog Devices, USA)	Easy to use, low-cost, gain range with 1 external resistor 1 to 10,000, low-noise (9 nV/√Hz @ 1 kHz), input voltage noise (0.28, ac characteristics: μV p-p noise (0.1 Hz to 10 Hz)), 120 kHz bandwidth (G = 100).	Suppressing the common-mode noises	Lab VIEW software (National Instruments, USA)	[18]
NI USB-6363 (National Instruments, USA)	Sample rate (max for single channel (2.00 M Sample/s), max for multi-channel (aggregate) (1.00 M Sample/s)), resolution timing (10 ns), accuracy timing (50 ppm), max working voltage for the analog inputs (signal + common mode) (±11 V), CMRR (DC to 60 Hz) 100 dB.	Digitization of acquired signals for converting analog to digital	Lab VIEW software (National Instruments, USA)	[18]
Instrumentation amplifier: AD622 (Analog Devices, USA)	Easy to use, low cost solution, gain range with 1 external resistor 2 to 2000, Unity gain with no external resistor, 66 dB (min CMRR) (G = 1), low noise (12 nV/√Hz @ 1 kHz), input voltage noise (0.60 μV p-p noise (0.1 Hz to 10 Hz)) (G = 10), ac characteristics: 10 μs settling time to 0.1% G = 1 to 100, 800 kHz bandwidth: G = 10, 10, 1.2 V/μs slew rate.	Suppressing the common-mode noises	Matlab (Mathworks, Inc., USA)	[22]
Operational amplifier: TLC2272 (Texas Instruments, Inc., USA)	Low noise: 9 nV/√Hz at f = 1 kHz (typical), low-input bias current (1–60 pA), high-gain bandwidth: 2.2 MHz (typical), high slew rate: 3.6 V/µs (typical), low input offset voltage: 950 µV max at TA = 25 °C, output current (2.2 mA), min CMRR (70 dB), Offset Drift (2 uV/C), temperature range: 40 to 125/0 to 70 C.	Amplification and filtering	Matlab (Mathworks, Inc., USA)	[22]
AD8232 (Analog Devices, USA))	Typical low supply current (170 µA) (typical), CMRR-80 dB (dc to 60 Hz), high signal gain (G = 100) with the dc blocking capabilities, single-supply operation (2.0 V to 3.5 V).	Signal to acquire, amplify, and filtering	Matlab (Mathworks, Inc., USA)	[22]
OPA124 (Texas Instruments, USA)	Typical low input capacitance (1 pf), high input resistance (1013 Ω), low noise: 6 nV/√Hz at f = 10 kHz, low bias current: 1 pA (max), low offset: 250 mv (max), low drift: 2 mv/°C (max), high open-loop gain: 120 dB (min), high common-mode rejection (min): 100 dB.	Reducing the high input impedance effect	Matlab (Math-Works, Inc., USA)	[111,339]
MP150, BioPAC systems (Aero Camino, Goleta, CA 93117, USA)	Band-pass filter (0.5–35 Hz), no-load power consumption (<150 mW), output power (2 W) (max), output current (<120 (DCM)/200 (CCM) mA) (max), Internal high-voltage current source.	Amplification and digitization	Matlab (Math-Works, Inc., USA)	[340]
INA118 (Texas Instruments Inc., USA)	Low offset voltage: 50 µV (max), low drift: 0.5 µV/°C (max), low input bias current: 5 nA (max), high CMR-110 dB (min), supply range (±1.35 to ±18 V), low quiescent current: 350 µA.	Amplification	Matlab (Math-Works, Inc., USA)	[341]
LMP7702(Texas Instruments Inc., USA)	Input offset voltage: ±220 µV (max), input bias current: ±200 fA, The specified low-offset voltage of less than ±200 µV, input voltage noise: 9 nV/√Hz, CMRR: 130 dB, open- loop gain (130 dB), temperature Range (−40 °C to 125 °C), Unity-gain bandwidth: 2.5 MHz, supply current-1.5 mA, supply voltage range: 2.7 V to 12 V.	Reducing the high input impedance effect	Matlab (Math-Works, Inc., USA)	[341]

## Data Availability

Not applicable.
